# Single-cell profiling reveals the trajectories of natural killer cell differentiation in bone marrow and a stress signature induced by acute myeloid leukemia

**DOI:** 10.1038/s41423-020-00574-8

**Published:** 2020-11-25

**Authors:** Adeline Crinier, Pierre-Yves Dumas, Bertrand Escalière, Christelle Piperoglou, Laurine Gil, Arnaud Villacreces, Frédéric Vély, Zoran Ivanovic, Pierre Milpied, Émilie Narni-Mancinelli, Éric Vivier

**Affiliations:** 1 Aix Marseille University, CNRS, INSERM, Centre d’Immunologie de Marseille-Luminy, Marseille, France; 2 CHU Bordeaux, Service d’Hématologie Clinique et de Thérapie Cellulaire, Bordeaux, France; 3 Bordeaux University, Bordeaux, France; 4 Institut National de la Santé et de la Recherche Médicale, U1035 Bordeaux, France; 5 APHM, Hôpital de la Timone, Marseille-Immunopôle, Marseille, France; 6 Établissement Français du Sang Nouvelle Aquitaine, Bordeaux, France; 7 Innate Pharma Research Laboratories, Innate Pharma, Marseille, France

**Keywords:** NK cells, scRNASeq, AML, Immunology, Innate immunity

## Abstract

Natural killer (NK) cells are innate cytotoxic lymphoid cells (ILCs) involved in the killing of infected and tumor cells. Among human and mouse NK cells from the spleen and blood, we previously identified by single-cell RNA sequencing (scRNAseq) two similar major subsets, NK1 and NK2. Using the same technology, we report here the identification, by single-cell RNA sequencing (scRNAseq), of three NK cell subpopulations in human bone marrow. Pseudotime analysis identified a subset of resident CD56^bright^ NK cells, NK0 cells, as the precursor of both circulating CD56^dim^ NK1-like NK cells and CD56^bright^ NK2-like NK cells in human bone marrow and spleen under physiological conditions. Transcriptomic profiles of bone marrow NK cells from patients with acute myeloid leukemia (AML) exhibited stress-induced repression of NK cell effector functions, highlighting the profound impact of this disease on NK cell heterogeneity. Bone marrow NK cells from AML patients exhibited reduced levels of CD160, but the CD160^high^ group had a significantly higher survival rate.

## Introduction

Natural killer (NK) cells are large granular lymphocytes in the ILC family. NK cells are endowed with the capacity to kill stressed cells, such as infected cells and tumor cells, without specific prior activation, in humans and mice.^1^ NK cells express an array of inhibitory and activating receptors, the engagement of which regulates NK cell activation. Inhibitory receptors are essential for sensing decreases in or total absence of constitutively expressed self MHC-I molecules on target cells. Decreases in MHC-I expression reduce the strength of the inhibitory signals delivered to NK cells, rendering these cells more prone to activation.^2,3^ NK cell activation results from the engagement of activating receptors, such as the activating isoforms of Ly49 and KIRs, natural cytotoxicity receptors (NCRs), SLAM (signaling lymphocyte activating molecule)-related receptors, NKG2D and CD16, which can induce NK cell activation by initiating different signaling pathways.^4^ The NCR family is composed of three molecules: NKp30 (*NCR3*, CD337) and NKp44 (*NCR2*, CD336), which are expressed in humans, and NKp46 (*NCR1*, CD335), which is expressed in all mammals and is highly conserved between humans and mice. NKp46 is expressed mostly by NK cells and ILC1 cells but is also present on a small population of T lymphocytes and a subset of ILC3 cells (NCR^+^ ILC3 cells) in mucosa.^5–7^

NK cells are present in blood and in primary and secondary lymphoid organs, including the spleen, bone marrow, liver, lymph nodes, lungs, tonsils, skin, uterus, and gut.^8^ The NK cell compartment consists of several subsets differing in maturation status. Human NK cells are defined as CD3^−^ CD14^−^ CD19^−^ CD56^+^ lymphocytes. They can be classified into subsets on the basis of the intensity of cell surface CD56 expression. Two principal NK cell subsets are found in the bloodstream in healthy individuals: CD56^dim^ CD16^+^ NK cells and CD56^bright^ CD16^−^ NK cells.^9–11^ The phenotypic differences between these cells are associated with functional differences: CD56^bright^ CD16^−^ NK cells are less cytotoxic than CD56^dim^ CD16^+^ cells but produce greater amounts of cytokines in response to exposure to environmental cues, such as interleukin (IL)-12 and IL-18.^9^ We recently used high-throughput scRNAseq to investigate the heterogeneity of the NK cell compartment in the blood and spleen of humans and mice.^12^ We identified two subsets conserved in human and mouse blood and spleen: NK1 and NK2 cells. In humans, the CD56^dim^ NK1 subset had high levels of cytotoxic activity, whereas the transcriptome of CD56^bright^ NK2 cells was enriched in gene ontology (GO) terms relating to the secretion of cytokines and chemokines, consistent with previous findings. We also found two tissue-specific human NK subsets resident in the spleen, which we named hNK_Sp3 and hNK_Sp4. hNK_Sp3 cells resembled CD56^bright^ NK2 cells, whereas hNK_Sp4 cells seemed to be more mature than hNK_Sp3 cells and more closely resembled the NK1 subset.

Several studies have suggested that CD56^bright^ CD16^−^ cells can develop into CD56^dim^ CD16^+^ NK cells. Indeed, CD56^bright^ NK cells constitute the main NK cell population shortly after hematopoietic stem cell transplantation and subsequently disappear as CD56^dim^ cell levels rise, 3 months after transplantation.^13^ Purified CD56^bright^ NK cells cocultured in vitro with synovial fibroblasts display downregulation of CD56 and a phenotype consistent with that of CD56^dim^ NK cells.^14^ In vivo, adoptive transfer of CD56^bright^ NK cells into immunodeficient NOD-SCID recipient mice led to a CD56^dim^CD16^+^ phenotype in the blood, spleen, and lymph nodes 10 days later.^14^ Finally, CD56^bright^ NK cells have been shown to have longer telomere repeats than CD56^dim^ NK cells, suggesting that they are less mature than CD56^dim^ NK cells.^14^

In both humans and mice, the expansion of a particular population of NK cells, called “adaptive NK” or “memory NK” cells, has been described during cytomegalovirus (CMV) infection.^15^ These adaptive NK cells persist long after the contraction of the anti-CMV immune response and are capable of more powerful effector functions upon reinfection. Adaptive NK cells have also been described following the activation of NK cells by cytokines^16^ or exposure to hantavirus infection,^17^ herpes simplex virus 2 infection^18^ or influenza vaccination in humans.^19^ Adaptive NK cells express the cell surface activating receptor NKG2C in humans and also express the maturation marker CD57, and they lack many transcription factors and signaling proteins, including FCRγ, PLZF, Siglec-7, EAT-2, and SYK.^20–22^

Acute myeloid leukemia (AML) is a hematological malignancy characterized by bone marrow invasion of proliferative, clonal immature myeloid cells.^23^ Blast cells replace normal hematopoietic cells and exploit the normal bone marrow microenvironment to survive and to support disease expansion, also affecting immune cells. NK cells from AML patients display low levels of cell surface NCR expression, which is associated with impaired cytotoxicity.^24,25^ Conversely, higher levels of NKp30 and NKp46 on the cell surface have been linked to better outcomes.^26,27^ Downregulation of other activating receptors, including DNAM-1, NKG2C and 2B4^28–30^ and upregulation of inhibitory receptors, such as NKG2A,^28,31^ on the surface of NK cells from AML patients also contribute to the low cytotoxicity and IFN-γ secretion levels of these cells.^32^

Here, we used unsupervised scRNAseq and unsupervised graph-based clustering to investigate human bone marrow NK cell heterogeneity under physiological conditions and at AML diagnosis. As previously found for the blood and spleen, healthy bone marrow contained NK1 and NK2 cells and an additional CD56^bright^-like resident NK cell subset, NK0 cells, from which NK1 and NK2 cells may differentiate. Comparison of transcriptomic profiles between NK cells from healthy and AML-derived bone marrow further revealed that AML profoundly modified the bone marrow NK cell compartment. Thus, AML affects NK cells at the pathological site.

## Results

### High-throughput scRNAseq identifies adaptive NK cells in human bone marrow

**Fig. 1:**
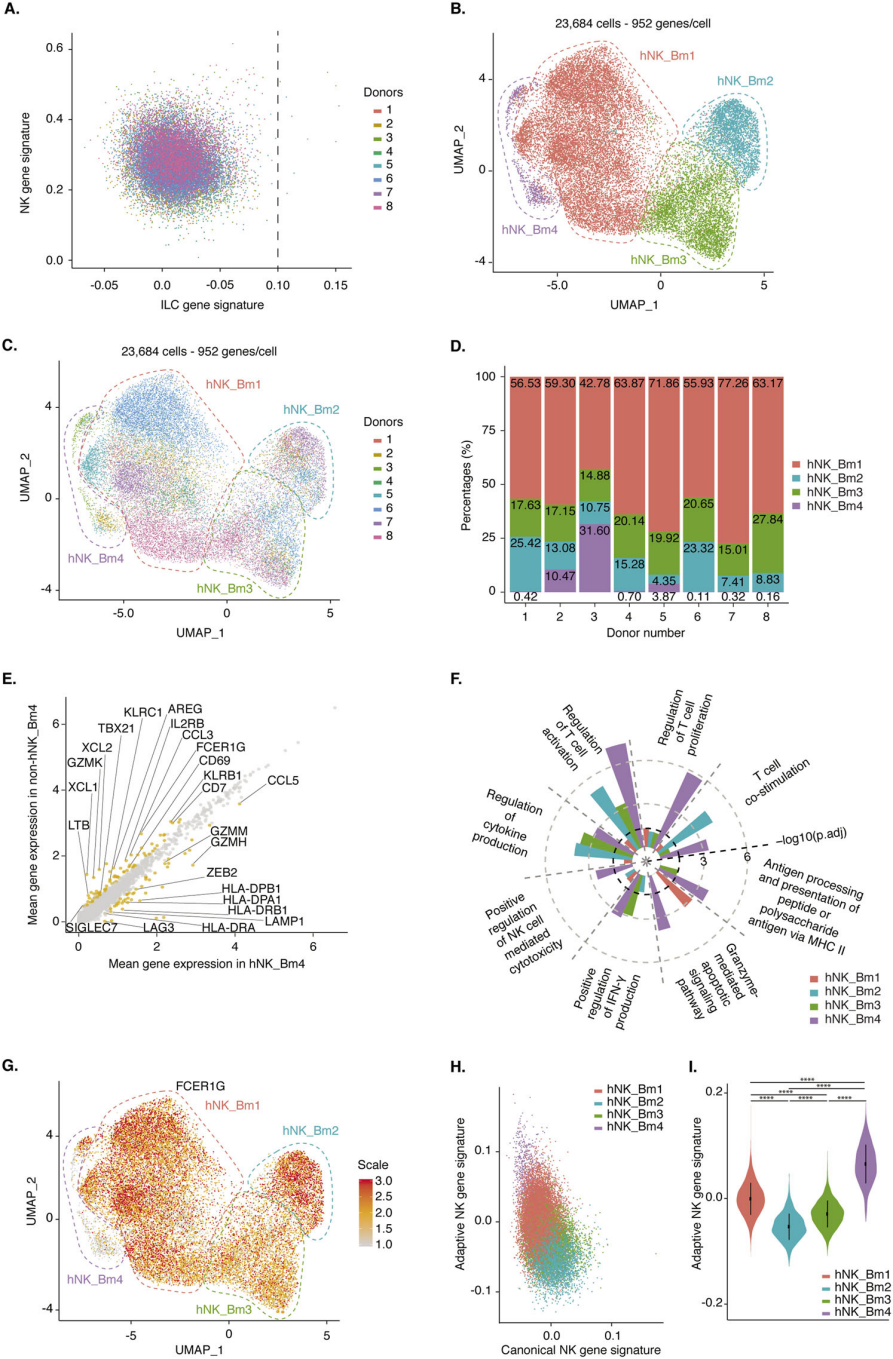
**A** Single-cell module score^10,34^ for each of the bone marrow NK cell samples sorted from eight different healthy donors for the NK and ILC gene signatures defined by^10^ and,^8^ respectively. Cells are color-coded according to donor origin. **B** UMAP of healthy human bone marrow NK cells from eight donors. Cells are color-coded according to the defined subset. **C** UMAP of 23,684 healthy human bone marrow NK cells from eight donors, identifying four subsets of NK cells. Cells are color-coded according to donor origin. **D** Percentage of each healthy bone marrow NK cell subset across the eight donors. Subsets are color-coded as above. **E** Scatter plot of the genes differentially expressed between NK cells from the hNK_Bm4 cluster and NK cells from the other three clusters. Genes displaying significant differential expression (*p* < 0.05) are represented by yellow dots, and selected genes are highlighted. **F** Selected Gene Ontology terms displaying enrichment in hNK_Bm4 cells. The Benjamini–Hochberg-corrected −log_10_ *p* value calculated by a hypergeometric test is reported. The black dotted line indicates the threshold of significance set at −log_10_(0.05). **G** Feature plot showing the relative expression levels of the *FCER1G* gene in each of the 23,762 healthy human bone marrow NK cells. **H** Module score for the CD56^dim^ CD57^+^ NKG2C^+^ adaptive and CD3^−^ CD56^dim^ CD57^−^ NKG2C^−^ canonical NK cell gene expression profiles obtained from reanalysis of the data of,^41^ for each of the human healthy bone marrow NK cells, at the single-cell level. **I** Violin plots showing the distribution of module scores for CD56^dim^ CD57^+^ NKG2C^+^ adaptive cells and for each blood NK cell grouped by subset. Statistical significance was assessed by the Kruskal–Wallis test with Dunn’s multiple comparison test, and *p* values were adjusted by the Benjamini-Hochberg method. *p value < 0.05, **p value < 0.01, ***p value < 0.001, ****p value < 0.0001

We investigated the heterogeneity of human bone marrow NK cells with the 10x Genomics high-throughput droplet-based scRNAseq pipeline, which can be used for unbiased transcriptomic characterization of thousands of cells simultaneously.^33^ We analyzed ~24,000 flow cytometry-sorted NK cells, defined by their SSC/FSC profile and as CD3^−^ CD14^−^ CD19^−^ CD45^+^ CD56^+^ cells, from the bone marrow of eight healthy donors. We verified that the transcriptomic profile of the sorted cells contained the core NK cell signature previously defined^12^ (data not shown). We confirmed that Lin^−^ CD56^+^ helper-like ILCs were absent from this population via module score analysis comparing single-cell human NK cell and helper-like ILC gene signatures, as previously described.^10,34^ This evaluation resulted in the removal of only ten contaminating helper-like ILCs from the ~24,000 cells analyzed (Fig. 1A). We analyzed bone marrow NK cell heterogeneity by projecting cells into two dimensions by uniform manifold approximation and projection (UMAP) analysis. UMAP analysis revealed the clustering of human bone marrow NK cells into four different subsets, hereafter referred to as hNK_Bm1, hNK_Bm2, hNK_Bm3, and hNK_Bm4 (Fig. 1B). The three main NK cell subsets (hNK_Bm1, hNK_Bm2, and hNK_Bm3) were common to all samples, whereas the hNK_Bm4 subset was absent from some donors (Fig. 1C,D). We nevertheless checked the accuracy of hNK_Bm4 cell identification by a machine learning approach (data not shown).

We first focused on hNK_Bm4 cells and identified 139 differentially expressed genes distinguishing this cluster from the other human bone marrow NK cell subsets: 88 of these genes were downregulated and 51 were upregulated in hNK_Bm4 cells (Fig. 1E and Supplementary Table S1A). We found that compared with the other human bone marrow NK cell subsets, hNK_Bm4 cells had lower expression levels of the *KLRB1* and *CD7* genes, encoding activating receptors; *KLRC1* (encoding NKG2A) and *SIGLEC7*, encoding inhibitory receptors; *IL2RB*, encoding the IL-2/IL-15Rβ (CD122) surface receptor essential for the development and differentiation of NK cells;^35^ and *FCER1G* (FcRγ) (Fig. 1E). By contrast, hNK_Bm4 cells had higher expression levels of genes encoding NK cell-associated effector proteins (*CCL5*, *GZMM*, and *GZMH)*^9,10^ and *ZEB2*, encoding a transcriptional regulator involved in terminal NK cell maturation.^36^ GO term analysis revealed that the transcriptome of hNK_Bm4 cells was specifically enriched in NK cell effector functions, such as positive regulation of interferon (IFN)-γ production, NK cell-mediated cytotoxicity and granzyme-mediated apoptotic signaling (Fig. 1F). The hNK_Bm4 subset also displayed upregulation of several genes relating to the class II major histocompatibility complex (MHC), T cell costimulation, and the regulation of T cell activation and proliferation (Fig. 1F). The hNK_Bm4 subset was identified in three of the five donors seropositive for human cytomegalovirus (HCMV) (Supplementary Table S2A). *FCER1G* downregulation (Fig. 1E,G) and GO term enrichment for antigen presentation and T cell activation (Fig. 1E) are other properties exhibited by adaptive NK cells.^37–40^ We therefore investigated whether the hNK_Bm4 subset could be considered to correspond to adaptive NK cells. We established canonical and adaptive NK cell gene signatures by analyzing the transcriptomic profiles of human blood CD3^−^ CD56^dim^ CD57^+^ NKG2C^+^ adaptive NK cells and CD3^−^ CD56^dim^ CD57^−^ NKG2C^−^ canonical NK cells from a public genome-wide bulk RNAseq dataset^41^ (Supplementary Fig. S1). Unsupervised hierarchical clustering (Supplementary Fig. S1A) and principal component analysis (PCA) (Supplementary Fig. S1B) segregated the samples into two groups corresponding to adaptive and canonical NK cells. A bilateral comparison of the two populations identified 912 genes among the total gene set as differentially expressed (Supplementary Table S1B). These genes included *KLRC2* (encoding NKG2C) (Supplementary Fig. S1C), which was overexpressed in adaptive NK cells. The expression of this gene is associated with the adaptive phenotype of NK cells during cytomegalovirus infection.^42^ The top 200 genes in both of these gene signatures intersected broadly with the genes detected in our scRNAseq dataset, which included 173 of the top 200 driver genes in the adaptive gene signature and 174 of the top 200 driver genes in the canonical gene signature (Supplementary Fig. S1D), indicating comparability. Module score analysis comparing the adaptive and canonical gene signatures of each of the bone marrow NK cell subsets revealed that the gene expression profile of hNK_Bm4 cells most closely resembled that of adaptive NK cells (Fig. 1H). Violin plots of the module scores among the subsets also revealed significant enrichment of the gene signature of adaptive NK cells in hNK_Bm4 cells (Fig. 1I). The hNK_Bm4 subset therefore had a transcriptomic profile different from that of conventional NK cells but closely resembling that of adaptive NK cells, suggesting that adaptive NK cells may populate the bone marrow of some HCMV-seropositive individuals.

### Three NK cell subsets are present in human bone marrow under physiological conditions

**Fig. 2:**
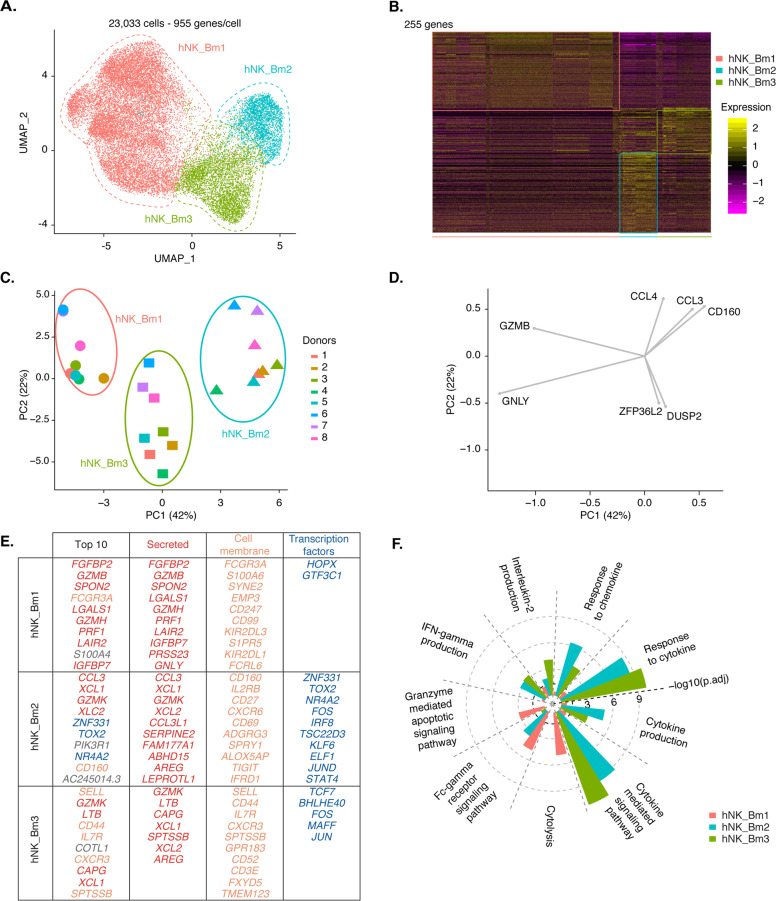
**A** UMAP of 23,033 healthy human bone marrow NK cells from eight donors, without hNK_Bm4, identifying three subsets of NK cells. Cells are color-coded according to the defined subset. **B** Heatmap of the 255 genes tested with the Wilcoxon rank sum test separating the 23,033 healthy human bone marrow NK cells into subsets. Cells are plotted in columns according to subset source. Genes are shown in rows and ranked by adjusted *p* values < 0.05. Gene expression is color-coded on a scale based on the *z*-score distribution, from −2.5 (purple) to 2.5 (yellow). The squares indicate specific transcriptomic signatures of human bone marrow NK cell subsets. **C** Principal component analysis on the three healthy human bone marrow NK cell subsets in each sample based on the mean expression levels of genes with differential expression. **D** Driver genes for each cell subset, accounting for 20% of the total variance for each PC. **E** Top ten most highly expressed genes among the total gene set and among genes encoding secreted proteins, cell membrane markers and transcription factors with significant differences among the three healthy human bone marrow NK cell subsets. Gene symbols and annotations were retrieved from public databases. Cell membrane protein-encoding genes are color-coded in orange, transcription factor-encoding genes in dark blue, secreted protein-encoding genes in red and genes encoding other proteins in gray. Genes are ranked by *p* value. **F** Selected Gene Ontology terms displaying enrichment in the three healthy human bone marrow NK cell subsets. The Benjamini–Hochberg-corrected −log_10_ *p* value calculated by a hypergeometric test is reported. The black dotted line indicates the threshold of significance set at −log_10_(0.05)

For further characterization of the heterogeneity of bone marrow NK cells among donors, we removed the hNK_Bm4 adaptive NK cell-like NK cell subset, which was absent from some donors, from the analysis. Unsupervised hierarchical clustering (data not shown) and UMAP analysis of the remaining ~23,000 human bone marrow NK cells again clustered the cells into three different subsets: hNK_Bm1, hNK_Bm2, and hNK_Bm3 (Fig. 2A). These three subsets were present in all samples (Fig. 1C, D). Significant differential expression between the hNK_Bm1, hNK_Bm2, and hNK_Bm3 subsets was observed for 255 of the genes composing the three different transcriptomic signatures of bone marrow NK cells (Fig. 2B, Supplementary Table S1C). No significant individual-specific phenotype or batch effect was observed in the unsupervised hierarchical clustering analysis (data not shown), and the NK subsets from a given individual did not cluster together on the PCA score plots (Fig. 2C). Instead, PC1 and PC2, accounting for 42% and 22% of the variance, respectively, and unsupervised hierarchical clustering (data not shown) segregated the eight donors into three different groups corresponding to the hNK_Bm1, hNK_Bm2, and hNK_Bm3 subsets (Fig. 2C). PC1 separated the hNK_Bm1, hNK_Bm2, and hNK_Bm3 subsets. The hNK_Bm3 subset was also separated from the hNK_Bm1 and hNK_Bm2 subsets by PC2. The *GNLY* and *GZMB* genes associated with cell cytotoxicity were identified as drivers for hNK_Bm1 along PC1. *CD160*, encoding an NK cell activating receptor, and *CCL3* and *CCL4*, encoding chemokines, were identified as drivers of the hNK_Bm2 subset, whereas *ZFP36L2* (which is involved in lymphocyte hematopoiesis^43,44^) and *DUSP2* (a regulator of the ERK pathway) were drivers of the hNK_Bm3 subset along PC1 and PC2 (Fig. 2D).

We then analyzed the top ten genes encoding secreted proteins, cell membrane markers and transcription factors expressed in hNK_Bm1, hNK_Bm2, and hNK_Bm3 cells (Fig. 2E). hNK_Bm1 cells displayed upregulation of genes associated with cytotoxic factors (*FGFBP2*, *GZMB*, *GZMH*, and *PRF1*) and overexpressed *FCGR3A* (encoding CD16), which is characteristic of the CD56^dim^ NK cell subset;^38–40^ moreover, these cells overexpressed *S1PR5*, which encodes a sphingosine 1-phosphate receptor promoting NK cell egress from the lymph nodes and bone marrow^45^ (Fig. 2E). The hNK_Bm2 subset transcriptome displayed enrichment with genes encoding soluble factors associated with NK cell effector functions (*CCL3*, *CCL4*, *XCL1*, *XCL2*, *GZMK,* and *CCL3L1*), amphiregulin (*AREG*), the activating receptor encoded by *CD160*, the activation marker encoded by *CD69*, and the chemokine receptor encoded by *CXCR6*, which is involved in ILC egress from the bone marrow^46^ (Fig. 2E). Like hNK_Bm2 cells, the hNK_Bm3 subset displayed higher expression levels of *GZMK*, *XCL1*, *XCL2*, and *AREG*, but these cells also displayed upregulation of the *LTB*, *SELL* (encoding CD62L) and *CD44* genes encoding adhesion molecules associated with homing and anchorage in the bone marrow^47,48^ (Fig. 2E). Biological process enrichment analysis revealed specific enrichment of the hNK_Bm1 subset transcriptome in the FcγR signaling pathway, the granzyme-mediated apoptotic signaling pathway and cytolysis (Fig. 2F). By contrast, the transcriptomes of both the hNK_Bm2 and hNK_Bm3 subsets displayed enrichment in chemokine and cytokine responses, cytokine production and cytokine-mediated signaling. The hNK_Bm2 subset transcriptome was more strongly enriched in responses, and the hNK_Bm3 subset transcriptome was more specifically enriched in IL-2 production. These results thus reveal the presence of three different subsets of human bone marrow NK cells under physiological conditions.

### The three human bone marrow NK cell subsets encompass an NK1-CD56^dim^-like subset, an NK2-CD56^bright^-like subset and an additional CD56^bright^-like subset

**Fig. 3:**
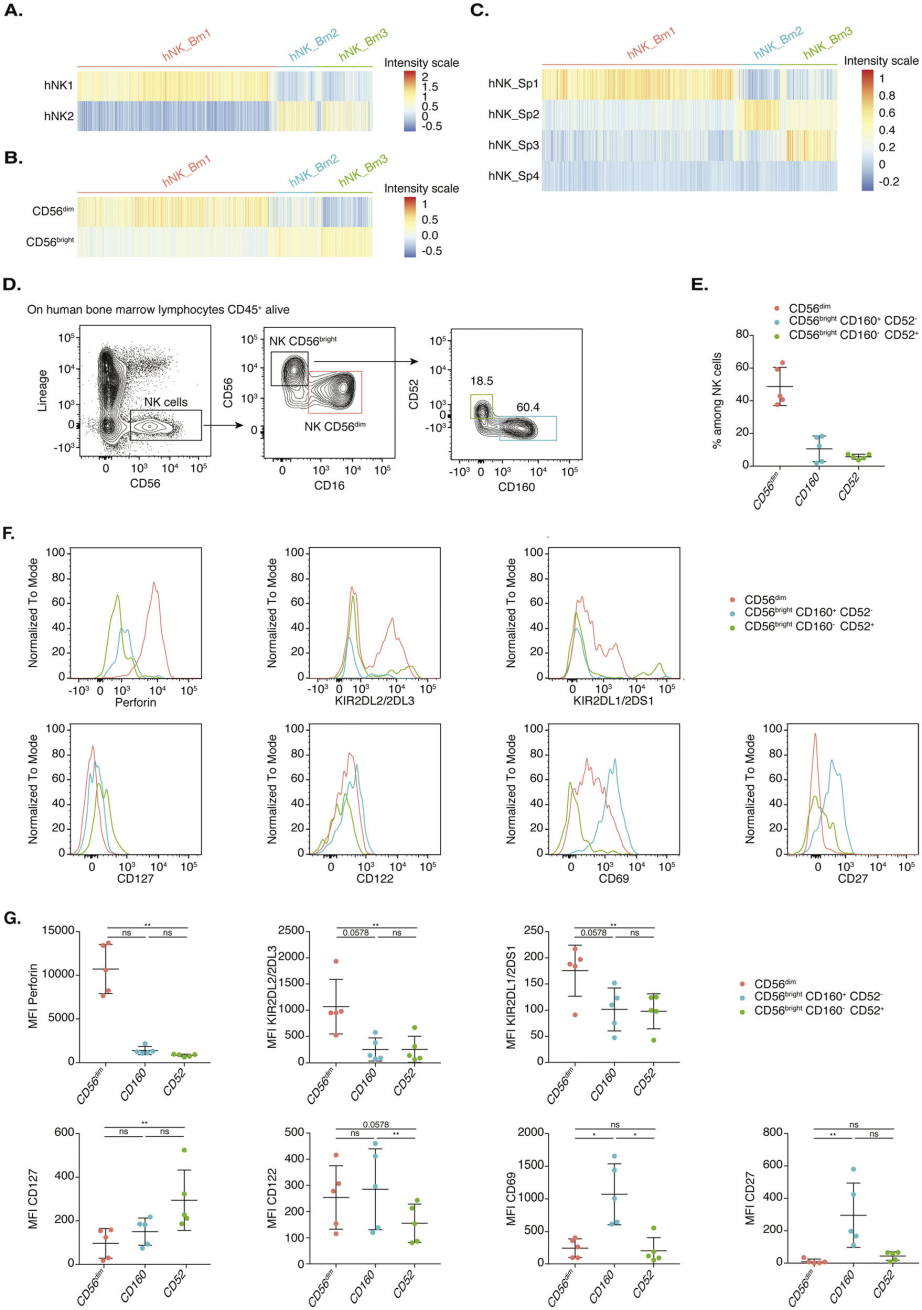
**A** Heatmap showing the hNK1 and hNK2 gene expression profiles of each of the three healthy human bone marrow NK cell subsets. **B** Heatmap showing the hNK_Sp1, hNK_Sp2, hNK_Sp3, and hNK_Sp4 gene expression profiles of each of the three healthy human bone marrow NK cell subsets. **C** Flow cytometric analysis of healthy bone marrow NK cells, showing CD52, CD160, and CD56 cell surface expression in one representative healthy donor. **D** Frequencies of the indicated cell populations among total bone marrow NK cells from five healthy donors. **E** FACS profiles of the indicated cell surface markers in the CD56^dim^, CD56^bright^ CD160^+^ CD52^−^, and CD56^bright^ CD160^−^ CD52^+^ cell subsets in one representative healthy donor. **F** Mean fluorescence intensity of the cell surface markers shown in **E** for each of the five healthy donors. Statistical significance was assessed by Friedmann’s analysis with Dunn’s post hoc test on paired subset data, and *p* values were adjusted by the Benjamini–Hochberg method. The error bars indicate the mean (±SD) values. *p value < 0.05, **p value < 0.01, ***p value < 0.001, ****p value < 0.0001

We then compared the transcriptomic signatures of the three human bone marrow NK cell subsets with those of the blood and splenic NK1 and NK2 subsets, which resembled CD56^dim^ and CD56^bright^ cells, respectively, and two additional spleen-specific hNK_Sp3 and hNK_Sp4 human NK cell populations, as previously described.^10,12^ Gene signature heatmaps (Fig. 3A–C) and module score analysis (Supplementary Fig. S2A–C) revealed that hNK_Bm1 cells corresponded to NK1, the related splenic hNK_Sp1 and CD56^dim^ NK cells, consistent with the pattern of *FCGR3A* expression. hNK_Bm2 corresponded to NK2, the related hNK_Sp2 and CD56^bright^ NK cells (Fig. 3A, B and Supplementary Fig. S2A–C). Further analysis revealed that the hNK_Bm3 subset shared part of the NK2 gene signature but was most similar to the minor hNK_Sp3 subset residing in the spleen (Fig. 3A, C and Supplementary Fig. S2C). No hNK_Sp4 subset signature was found in bone marrow NK cells (Fig. 3C).

Flow cytometric analysis of bone marrow NK cells revealed that the expression of CD160 and CD52 (CAMPATH-1) on the surface of CD56^bright^ cells was mutually exclusive, further discriminating between hNK_Bm2 and hNK_Bm3, respectively, as previously reported for hNK_Sp2 and hNK_Sp3 in the spleen^12^ (Fig. 3D). These analyses showed that the human bone marrow NK cell compartment consists mostly of CD56^dim^ NK cells (~50%), with a few CD56^bright^-like CD160^+^ CD52^−^ NK cells (~10%) and CD56^bright^ CD160^−^ CD52^+^ NK cells (~5%) (Fig. 3E). The protein levels of perforin, KIR2DL2/2DL3 and KIR2DL1/2DS1 were higher in CD56^dim^-like cells than in CD56^bright^-like CD160^+^ CD52^−^ NK cells and CD56^bright^ CD160^−^ CD52^+^ NK cells, confirming our transcriptomic data (Fig. 3F, G). The hNK_Bm2 subset was characterized by higher levels of CD122, CD27, and CD69, whereas hNK_Bm3 cells had higher levels of CD127 (Fig. 3F, G).

Together, these results demonstrate that the hNK_Bm1 and hNK_Bm2 subsets resemble the NK1-CD56^dim^ and NK2-CD56^bright^ subsets, respectively, which also reside in the blood and spleen in humans. The third subset, hNK_Bm3, most closely resembled the hNK_Sp3 population resident in the spleen and absent from the bloodstream.

### The NK0 subset has features consistent with a role as the precursor of both the NK2 and NK1 subsets

**Fig. 4:**
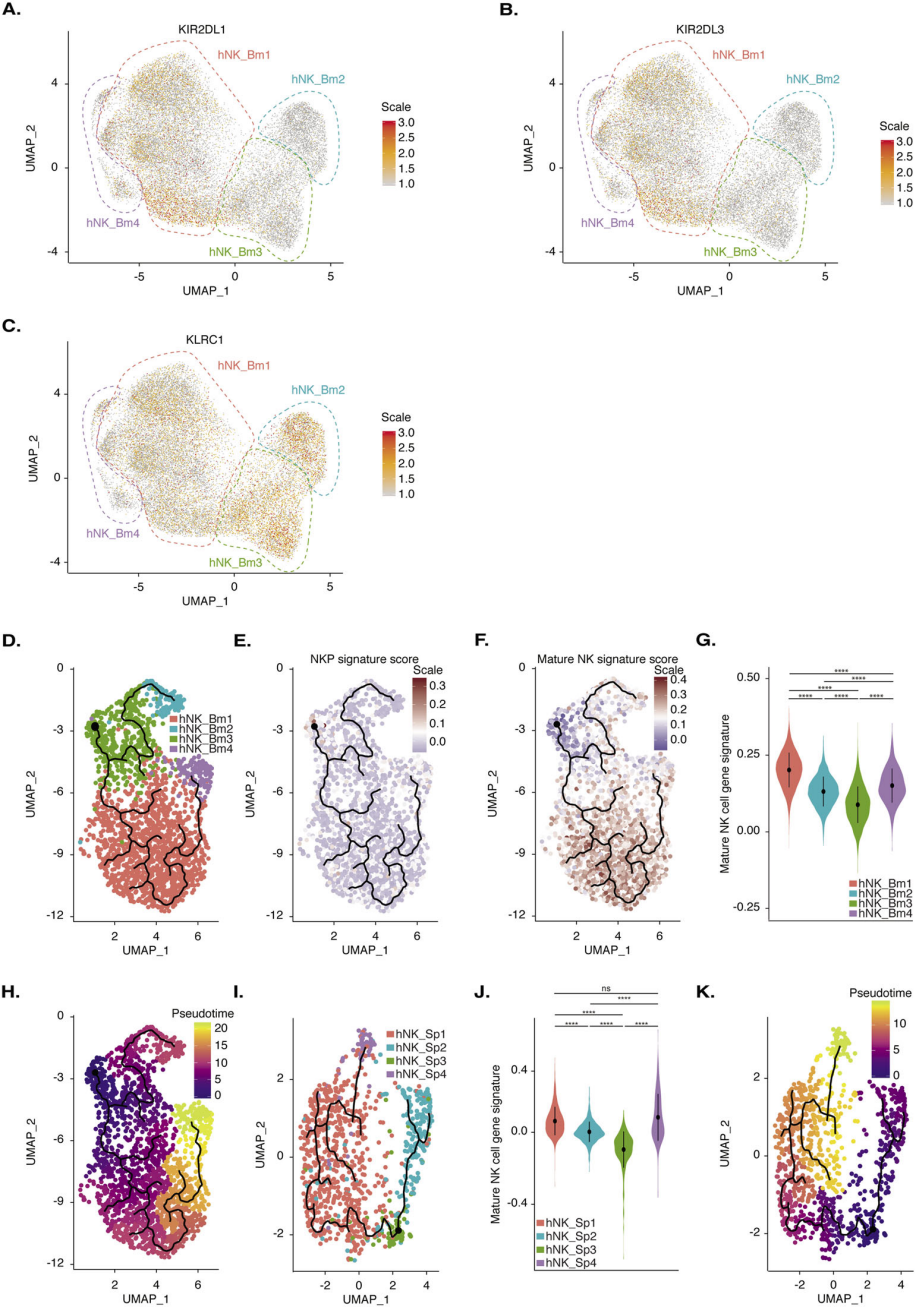
**A** Feature plot of the relative expression level of the *KIR2DL1* gene in each of the 23,066 healthy human bone marrow NK cells. **B** Feature plot of the relative expression level of the *KIR2DL3* gene in each of the 23,066 healthy human bone marrow NK cells. **C** Feature plot of the relative expression level of the *KLRC1* gene in each of the 23,066 healthy human bone marrow NK cells. **D** Pseudotime inference of the bone marrow NK cell trajectory in one representative healthy donor from the eight studied donors, with cells color-coded according to the corresponding bone marrow NK cell subset identified by UMAP. **E** Module score for the CD34^+^ CD38^+^ CD123^−^ CD45RA^+^ CD7^+^ CD10^+^ CD127^−^ bone marrow NKP gene expression profile obtained by reanalysis of the data of^51^ for one representative healthy donor by the pseudotime algorithm. **F** Module score for the CD3^−^ CD56^+^ NKp46^+^ bone marrow mature NK cell gene expression profile obtained by reanalysis of the data of^51^ for one representative healthy donor by the pseudotime algorithm. **G** Violin plot showing the distribution of the module scores for CD3^−^ CD56^+^ NKp46^+^ mature NK cells among each human healthy bone marrow NK cell subset, grouped by subset. **H** Pseudotime inference by the pseudotime algorithm from an starting point selected as the most distal cell of the hNK_Bm3 subset in one healthy donor representative of the eight studied donors. The scale indicates the maturation state, from dark blue (least mature) to yellow (most mature). **I** Pseudotime inference of the splenic NK cell trajectory in one healthy donor representative of the three studied donors, with cells color-coded according to the corresponding splenic NK subset identified by UMAP, as previously described by.^12^ **J** Violin plot showing the distribution of the module scores for CD3^−^ CD56^+^ NKp46^+^ mature NK cells among each human healthy splenic NK cell subset, grouped by subset. **K** Pseudotime inference by the pseudotime algorithm from an starting point selected as the most distal cell of the hNK_Sp3 subset in one healthy donor representative of the three studied donors. The scale indicates the maturation state, from dark blue (least mature) to yellow (most mature)

We then investigated whether the four NK cell subsets identified in the bone marrow reflected differences in maturation status. *KLRC1* was expressed at higher levels in the hNK_Bm2 and hNK_Bm3 subsets, whereas expression of the *KIR2DL3* and *KIR3DL1* killer cell immunoglobulin-like receptor (KIR) genes was restricted to the hNK_Bm1 subset (Fig. 4A–C). These results suggest that the hNK_Bm1 subset is more mature than the hNK_Bm2 and hNK_Bm3 subsets because a decrease in cell surface expression of NKG2A and a concomitant increase in KIR expression have been associated with human NK cell differentiation.^49^ We then searched for a developmental path across bone marrow NK subsets by applying the pseudotime algorithm Monocle DDRTree, which computationally orders the transcriptomic profiles of cells along a trajectory without prior information about their clustering.^50^ The pseudotime algorithm ordered the cells along a trajectory segregating our ~24,000 bone marrow NK cells into two branches and four clusters. These four clusters overlapped perfectly with the hNK_Bm1, hNK_Bm2, hNK_Bm3, and hNK_Bm4 subsets defined by UMAP, providing additional support for our previous conclusions. The hNK_Bm3 subset was located at the intersection of two branches linking the hNK_Bm1-hNK_Bm4-hNK_Bm3 and hNK_Bm2-hNK_Bm3 subsets (Fig. 4D).

We then tried to define the starting point of the putative developmental trajectory by comparing our dataset with an NK cell precursor (NKP) gene signature. We examined the transcriptomic profiles of bone marrow Lin^−^ CD34^+^ CD38^+^ CD123^−^CD45RA^+^ CD7^+^ CD10^+^ CD127^−^ NKP and CD3^−^ CD56^+^ NKp46^+^ mature NK cells from a public genome-wide RNAseq dataset.^51^ Unsupervised hierarchical clustering separated the samples into two main branches on the basis of a precursor or a mature NK cell phenotype, suggesting that these two populations can be differentiated on the basis of a specific transcriptomic pattern (Supplementary Fig. S3A). PCA confirmed this finding, as mature NK cells clustered away from progenitors along a combined PC1 and PC2 axis, accounting for 30.7% and 16.6% of the total variance, respectively (Supplementary Fig. S3B). Bilateral comparison of the two populations identified 448 genes from the total gene set as differentially expressed, with 265 upregulated in NK cells and 183 upregulated in progenitors (Supplementary Fig. S3C, Supplementary Table S1D). Mature NK cells had increased levels of *CD160*, *FCGR3A*, *GZMB*, and *CCL3* expression, whereas NK cell progenitors displayed upregulation of *TCF4* and *RAG2*, which are expressed during NK cell development^52^ (Supplementary Fig. S3C). These gene signatures broadly intersected with the genes detected by our scRNAseq analysis, with 263 genes from the mature NK cell signature and 160 genes from the progenitor gene signature found in our dataset (Supplementary Fig. S3D). Module score analyses comparing the NKP and mature NK cell gene signatures among bone marrow NK cell subsets were performed on the developmental trajectory defined by the pseudotime algorithm (Fig. 4E, F). These analyses revealed that the hNK_Bm3 subset displayed specific enrichment for the NKP signature, whereas the hNK_Bm1 and hNK_Bm4 subsets were enriched for the mature NK cell profile (Fig. 4E, F). Violin plots of the NK gene signature module scores also showed that the hNK_Bm3 subset was the least mature (Fig. 4G). The hNK_Bm3 subset was, therefore, defined as the starting point of the pseudotime developmental trajectory (Fig. 4H).

Pseudotime analysis with Monocle DDRTree revealed a specific developmental trajectory of human bone marrow NK cells, with the CD56^bright^-like hNK_Bm3 minor subset differentiating into the NK1-CD56^dim^-like hNK_Bm1 subset and a distinct NK2-CD56^bright^-like hNK_Bm2 subset (Fig. 4H). The NK1-CD56^dim^-like hNK_Bm1 and NK2-CD56^bright^-like hNK_Bm2 subsets followed different developmental pathways. hNK_Bm4, the adaptive NK cell subset, appeared to arise at a later time point from the hNK_Bm1 subset (Fig. 4H). Similar results were obtained with another pseudotime algorithm (PAGA Tree from Dynverse), providing additional support for our putative differentiation trajectory (data not shown).

In addition to the bone marrow, we reanalyzed our previous splenic dataset^12^ with the recently developed pseudotime algorithm Monocle DDRTree. Pseudotime analysis ordered the splenic NK cells into two branches and four clusters corresponding to the hNK_Sp1, hNK_Sp2, hNK_Sp3, and hNK_Sp4 subsets (Fig. 4I). The hNK_Sp3 subset was identified as the least mature, with the hNK_Sp2, hNK_Sp1, and hNK_Sp4 subsets sequentially more mature (Fig. 4J). Thus, in the spleen, as in the bone marrow, a minor CD56^bright^-like hNK_Sp3 subset differentiates into the NK1-CD56^dim^-like hNK_Sp1 subset and a distinct NK2-CD56^bright^-like hNK_Sp2 subset (Fig. 4K). Thus, hereafter, we refer to the CD56^bright^-like hNK_Bm3 and CD56^bright^-like hNK_Sp3 subsets as NK0 (Supplementary Fig. S4). The hNK_Sp4 subset, an additional minor CD56^dim^ splenic NK cell subset for which no corresponding subset was found in the bone marrow, seemed to differentiate from the NK1-CD56^dim^-like hNK_Sp1 subset.

### Acute myeloid leukemia affects the transcriptome of human bone marrow NK cells

**Fig. 5:**
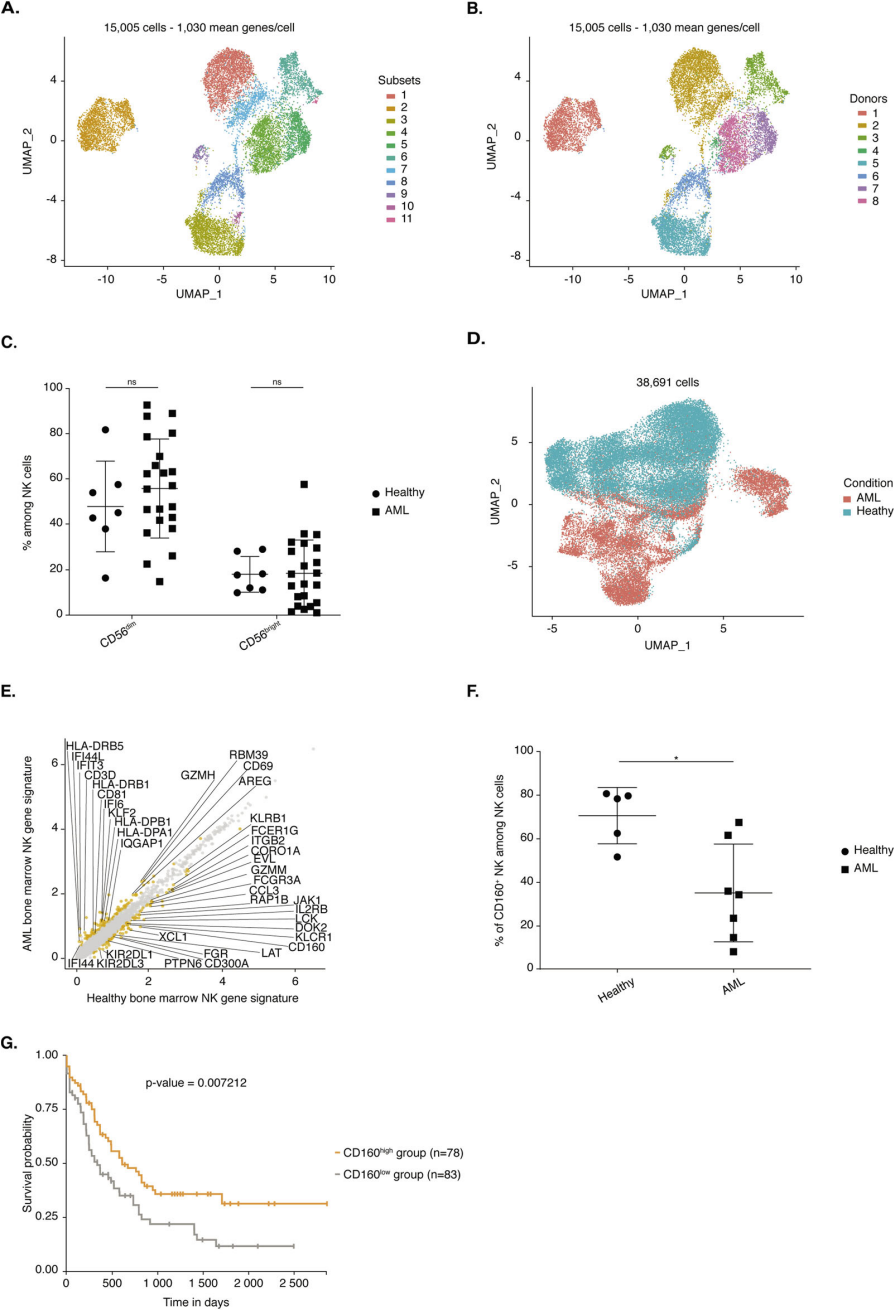
**A** UMAP of 15,005 human bone marrow NK cells from eight donors with AML. Cells are color-coded according to the defined subset. **B** UMAP of 15,005 human bone marrow NK cells from eight donors with AML. Cells are color-coded according to donor origin. **C** Frequencies of CD56^bright^ and CD56^dim^ cells among CD3^−^ CD56^+^ or CD3^−^ CD14^−^ CD19^−^ CD56^+^ cells from seven healthy donors and 22 donors with AML from the pooled data of two independent experiments. **D** UMAP of 38,691 human bone marrow NK cells from eight healthy donors and eight donors with AML. Cells are color-coded according to the donor’s clinical status. **E** Scatter plot of the genes differentially expressed between healthy and AML NK cells. Genes displaying significant differential expression (*p* value < 0.05) are represented by yellow dots, and selected genes are highlighted. **F** Frequencies of CD160-expressing cells among the total CD3^−^ CD14^−^ CD19^−^ CD56^+^ NK cell population from five healthy donors and seven donors with AML. Statistical significance was assessed by Friedmann’s analysis with Dunn’s post hoc tests on paired subset data, and *p* values were adjusted by the Benjamini–Hochberg method. The error bars indicate the mean (±SD) values. *p value < 0.05, **p value < 0.01, ***p value < 0.001, ****p value < 0.0001. **G** Kaplan–Meier overall survival curves for AML patients from TCGA stratified by the *CD160* expression level. The optimal cutoff for patient stratification was obtained with a Cox proportional hazards model, and the *p* value was calculated by the log-rank test. *CD160*^high^ group (≥7.427, *n* = 78); *CD160*^low^ group (<7.427, *n* = 83)

Acute myeloid leukemia (AML) is a specific bone marrow disease known to be associated with strong impairment of NK cell function in the periphery.^27^ We therefore investigated the impact of AML on the heterogeneity of bone marrow NK cells. We analyzed ~15,000 flow cytometry-sorted bone marrow NK cells, defined as CD3^−^ CD14^−^ CD19^−^CD34^−^ CD56^+^ cells, obtained from eight AML patients at diagnosis (Supplementary Table S2B). Module score analysis comparing human NK cell and helper-like ILC gene signatures resulted in the removal of 39 ILC-like cells (Supplementary Fig. S5A). UMAP analysis on the remaining cells revealed the existence of several NK cell subsets (Fig. 5A), but their assignment on the basis of donor origin showed that these clusters had a strong donor phenotype (Fig. 5B). There was no conserved NK cell subset in the AML patients (data not shown). Moreover, there was no conserved NK cell subset when AML patients were considered according to the same French–American–British classification of AML (data not shown). We then investigated whether module score analysis could be used to compare the specific signatures of each of the hNK_BM subsets to this AML dataset (Supplementary Fig. S5B–E). Module score analysis was performed on the UMAP representation of total NK cells from the AML patients (Supplementary Fig. S5B–E). A high level of transcriptomic heterogeneity was observed at the patient level for both subset composition and percentage. Based on protein levels, we detected heterogeneity in the percentages of the CD56^bright^ and CD56^dim^ subsets among the AML samples (Fig. 5C). Thus, AML strongly affected the transcriptomic profile of bone marrow NK cells, making it impossible to analyze their heterogeneity with classical annotation tools.

We therefore further investigated the effects of AML on bone marrow NK cells by analyzing a mixture of bone marrow NK cells from healthy donors and AML patients. UMAP analysis of the ∼38,700 bone marrow NK cells revealed that NK cells clustered differentially according to the clinical status of the donor (Fig. 5D). In total, 197 genes were differentially expressed between bone marrow NK cells from healthy donors and those from donors with AML: 90 genes were upregulated in NK cells from healthy donors, and 107 genes were upregulated in NK cells from donors with AML (Figs. 5E, Supplementary Figs. S6 and S7 and Table S1E). Bone marrow NK cells from AML patients had higher expression levels of interferon-induced genes (*IFI44L*, *IFI6*, *IFIT3,* and *IFI44*), HLA molecule-encoding genes (*HLA-DPB1*, *HLA-DPA1*, *HLA-DRB5*, and *HLA-DRB1*), *ZEB2*, and *KLF2*, which encode transcription factors controlling NK cell maturation^36^ and survival,^53^ respectively. By contrast, NK cells from the bone marrow of healthy donors displayed higher expression levels of genes encoding NK cell effector molecules (*CCL3*, *CCL4* and *GZMM*) and genes encoding surface receptors (CD16 (*FCGR3A*), CD161 (*KLRB1*), NKG2A (*KLRC1*), *CD300A*, FcγR (*FCER1G*), *KIR2DL3*, *KIR2DL1,* and *CD160*) (Fig. 5E and Supplementary Fig. S6 and Table S1E). The higher level of CD160 expression in bone marrow NK cells from healthy donors than in bone marrow cells from donors with donors with AML was confirmed by flow cytometric assessment of protein levels (Fig. 5F). We then further investigated the potential role of CD160 in AML pathogenesis and progression by studying the clinical outcomes of cancer patients in the TCGA database. The survival rates of patients with CD160^high^ AML were much higher than those of patients with CD160^low^ cancer, suggesting that CD160 is an antitumor biomarker in AML (Fig. 5G). In addition, bone marrow NK cells from healthy donors displayed stronger expression of genes involved in regulating NK cell effector functions (*DOCK2*, *EVL*, *LCK*, *RAP1B*, *LAT*, *JAK1*, *CORO1A,* and *FGR*^54–61^), *PTPN6* (encoding SHIP-1), and *ITGB2*, which has been reported to promote the NK cell response^62,63^ (Fig. 5E and Supplementary Fig. S7 and Table S1E).

**Fig. 6:**
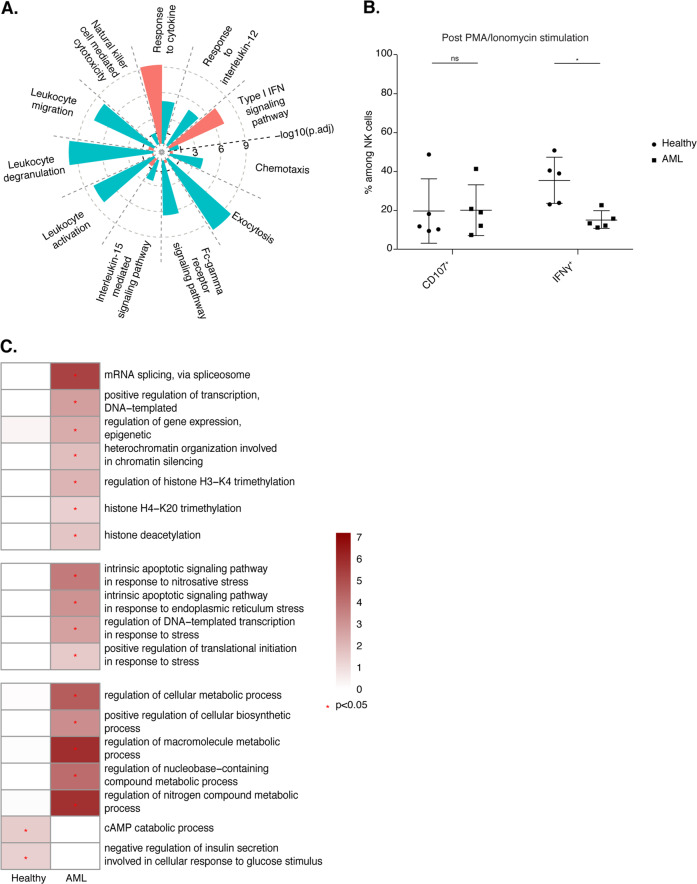
**A** Selected Gene Ontology terms displaying enrichment in healthy and AML samples. The Benjamini−Hochberg-corrected −log_10_ *p* value calculated by a hypergeometric test is reported. The black dotted line indicates the threshold of significance set at −log_10_(0.05). **B** Frequencies of CD107^+^ and IFN-γ^+^ cells among CD3^−^ CD56^+^ NK cells from five healthy donors and five donors with AML from the pooled data of two independent experiments. Statistical significance was assessed by Friedmann’s analysis with Dunn’s post hoc tests on paired subset data, and *p* values were adjusted by the Benjamini–Hochberg method. The error bars indicate the mean (±SD) values. *p value < 0.05, **p value < 0.01, ***p value < 0.001, ****p value < 0.0001. **C** Heatmap of selected Gene Ontology terms related to gene expression, the stress response and metabolism. The Benjamini–Hochberg-corrected −log_10_ *p* values calculated by a hypergeometric test are reported on a scale from 0 to 6. The red star indicates the threshold of significance set at −log_10_(0.05)

Biological process enrichment analysis revealed enrichment in the GO terms “response to cytokine” and “type I interferon signaling pathways” in bone marrow NK cells from AML patients (Fig. 6A) relative to bone marrow NK cells isolated from healthy individuals. By contrast, NK cells purified from healthy bone marrow displayed enrichment in the GO terms “NK cell-mediated cytotoxicity”, “FcγR signaling pathway”, “exocytosis”, “response to IL-12” and “IL-15-mediated signaling pathways”. Collectively, these results suggest that bone marrow NK cells from healthy donors have a more activated phenotype than those isolated from AML patients, which displayed a strong type I IFN response signature. Indeed, upon simulation with phorbol myristate acetate and ionomycin, higher frequencies of IFN-γ-producing NK cells were detected in healthy donors than in AML patients, consistent with the above findings (Fig. 6B). We extended our analysis of NK cell transcriptomic profiles further by assessing the enrichment in GO terms related to cell metabolism (Fig. 6C). Relative to bone marrow NK cells from healthy individuals, bone marrow NK cells from AML patients displayed enrichment in GO terms associated with the regulation of gene expression through the processes of transcription, splicing, and epigenetic modifications; the regulation of macromolecules; and metabolic processes and stress-related signaling. These results highlight the impact of AML on bone marrow NK cells.

## Discussion

The main objective of this study was to address, from an unbiased transcriptome-wide perspective, the heterogeneity of NK cells in human bone marrow under physiological conditions and at the time of diagnosis of acute myeloid leukemia, a hematologic malignancy of the bone marrow.

We report here four different clusters of Lin^−^CD56^+^ cells in bone marrow—hNK_Bm1, hNK_Bm2, hNK_Bm3, and hNK_Bm4—the last of which was not present in all donors. The unsupervised hierarchical clustering results were further validated by machine learning algorithms, supporting the robustness of our analysis. By contrast, scRNAseq profiling of Lin^−^ CD7^+^ cells from healthy human bone marrow identified six NK cell clusters,^64^ with CD7 expressed by both progenitor and mature ILCs.^65^ Our analysis of Lin^−^ CD56^+^ cells was thus expected to yield different results. However, similarities may exist between the two studies, as the ‘mature’ and ‘terminal’ populations characterized by *PRF1*, GZMB, *S100A6*, *FCGR3A*, *LGALS1*, and *SPON2* expression^64^ resembled the hNK_Bm1 subset; the “active” and “transitional” populations expressing *CCL3, XCL2, PI3KR1, IRFD1*, and *FOS*^64^ resembled the hNK_Bm2 subset; and the CD56^bright^ subset^64^ resembled the CD56^bright^ hNK_Bm3 subset, with the expression of *IL7R*, *CD44*, *LTB*, *XCL1*, *GZMK*, *SELL*, and *XCL2*.

We identified three subsets of NK cells, including the conserved NK1-CD56^dim^-like and NK-CD56^bright^-like subsets, in the bone marrow of all healthy humans. Both the NK1 and NK2 subsets are, therefore, present in the blood, spleen, and bone marrow. This finding is consistent with the origination of NK cells in the bone marrow and with their release into the bloodstream and continual recirculation throughout the body, leading to the population of several organs, including the spleen.^9,11^ Consistent with our previous findings,^12^ we observed that the NK1-CD56^dim^ subset had a transcriptomic profile enriched in markers of cytotoxic activity, whereas the transcriptomic profile of NK2-CD56^bright^ cells was more heavily enriched in genes involved in the response to cytokines and chemokines. Preliminary data indicated that NK1 and NK2 cells were also present in nonlymphoid organs, such as the duodenum and lungs, but these findings require further exploration.

We detected an additional NK0 CD56^bright^-like subset, originally identified as hNK_Bm3, which resembled the hNK_Sp3 subset residing in the human spleen.^12^ NK0 cells were not found in the blood, explaining the previous lack of detection of this subset. The cells of this population expressed high surface levels of CD56 (CD56^bright^) and had a transcriptomic signature similar to that of the CD56^bright^ NK subset but could be distinguished from NK2 cells on the basis of their higher levels of CD52 and CD127 expression and lower levels of CD160 expression. Additional experiments are necessary to clarify the real relationship between the hNK_Bm3 subset and the conventional CD56^bright^ NK subset. We found that the transcriptome of NK0 cells displayed specific enrichment with genes associated with the NK cell precursor (NKP) signature. The pseudotime algorithm Monocle DDRTree, which infers developmental trajectories from scRNAseq data, showed that bone marrow NK0 (hNK_Bm3) cells can differentiate into both NK1 and NK2 cells (Supplementary Fig. S4). Our data thus show that a minor subset of the CD56^bright^ NK cell population, the subpopulation of tissue-resident CD56^bright^ CD127^+^ CD160^−^ CD52^+^ cells (referred to hereafter as NK0 cells) can give rise to other NK2/CD56^bright^ CD160^+^ CD52^−^ cells and to NK1/CD56^dim^-perforin^high^ cells under physiological conditions. These results are consistent with those of previous studies showing that CD56^bright^ NK cells can differentiate into CD56^dim^ NK cells^13^ and provide the first demonstration, at the transcriptomic level, of such differentiation in humans in vivo. NK1 and NK2 cells appeared at the end of the two branches on the pseudotime trajectory, suggesting that these two populations are not developmentally related. This observation may explain why not all CD56^bright^ cells differentiate into CD56^dim^ cells.^14^ However, upon cytokine-induced activation,^66^ coculture with synovial fibroblasts or transfer into NOD/SCID mice,^14^ CD56^bright^ NK2-like cells can differentiate into CD56^dim^ NK cells. These results thus suggest that under certain circumstances, circulating CD56^bright^ NK2-like NK cells in peripheral blood can also give rise to CD56^dim^-like cells. However, it remains to be determined whether this phenomenon naturally occurs without external manipulation and to what extent the CD56^dim^-like subset generated resembles the conventional CD56^dim^ subset.

Similar analyses of the human spleen showed that splenic NK0 (hNK_Sp3) cells can also differentiate into both NK1 and NK2 cells. It was long supposed that NK cells develop exclusively within the bone marrow, but it is now clear that there is enrichment of some NK cell precursors in extramedullary tissues.^67^ The biological relevance of extramedullary NK cell differentiation and the mechanisms governing this process remains to be determined.

In this study, we also used public datasets containing transcriptomic profiles of canonical and adaptive NK cells^51^ to define the first transcriptomic signature of human adaptive NK cells. Based on transcriptomic data, we detected adaptive NK cells in the bone marrow of human CMV-seropositive individuals. The presence of adaptive NK cells was correlated with CMV seropositivity, but not all CMV-seropositive donors had detectable (at least by transcriptome analysis) adaptive NK cell subsets. These results were consistent with those of a previous study by Schlums and coworkers showing that not all CMV-seropositive individuals have circulating adaptive NK cells.^40^ Thus, adaptive NK cells appear to be present in the bone marrow, in addition to the blood and spleen.

A population of tissue-resident NK cells, ltNK cells, has recently been described.^68^ These cells are absent from the blood but can account for ~29% of NK cells in the bone marrow, ~45% of splenic NK cells and ~56% of NK cells in lymph nodes.^68^ ltNK cells have a CD69^+^ CXCR6^+^ cell surface phenotype but disparate levels of CD56 and CD16,^68^ suggesting that this population, at least the CD56^bright^ and CD56^dim^ subsets, is heterogeneous. The transcriptomes of the hNK_Bm3 and hNK_Sp3 subsets, corresponding to our NK0 tissue-resident progenitors, were not enriched in the transcriptomic signature of ltNK cells defined by bulk RNAseq on flow cytometry-sorted CD69^+^ CXCR6^+^ cells.^69^ Given that in RNAseq, the gene expression profile of the considered population is averaged and that ltNK cells have a heterogeneous surface phenotype, these cells may also have a heterogeneous transcriptomic profile, preventing their identification at the transcriptomic level by scRNAseq.

We tried to explore the heterogeneity of the bone marrow NK cell compartment in AML patients at diagnosis. We found that each sample had a unique specific transcriptomic profile, preventing subset assignment. A similar patient-specific pattern was also recently observed for the expression of NKp30 and CD27 on the surface of NK cells from AML patients.^70^ We were nevertheless able to extract a transcriptomic signature of NK cells under AML disease conditions relative to physiological conditions. Bone marrow NK cells from AML patients had a transcriptomic profile enriched in genes involved in responses to cytokine and type I interferon signaling pathways. Consistent with the well-known impairment of NK cell function during AML,^24,25,27^ NK cells isolated from healthy individuals had a transcriptomic profile enriched in genes involved in cell cytotoxicity, the FcγR receptor signaling pathway, exocytosis, and the response to IL-12 and IL-15 signaling pathways, indicating a more activated NK cell phenotype for these cells than for those isolated from AML patients. Indeed, higher percentages of bone marrow NK cells from healthy donors produced IFN-γ upon PMA/ionomycin stimulation than did than bone marrow NK cells from AML patients. In addition, the impairment of NK cells from AML patients at the site of disease onset was consistent with the capacity of AML blasts to affect NK cells in vitro.^31,71,72^ The exact mechanism by which tumor cells affect NK cells remains to be determined.

Bone marrow from AML cancer patients also exhibited downregulation of mRNA expression of *CD160*, whose protein expression was also found to be downregulated. Moreover, among AML patients, higher CD160 levels correlated with better survival, suggesting that CD160 could be a marker of interest in AML. This glycoprotein activates the effector functions of NK cells.^73^ However, the biological relevance of CD160 signaling at the surface of NK cells in the context of AML awaits further investigation.

Overall, our study identified an NK0 CD56^bright^-like subset as the precursor of the NK1-CD56^dim^ and NK2-CD56^bright^ subsets. In addition, our findings regarding AML revealed that NK cells undergo donor-specific cancer-associated editing and provided further evidence of profound NK cell impairment in the bone marrow at diagnosis.

## Experimental model details

### Samples

Frozen healthy bone marrow samples were recovered from filters used for allogeneic hematopoietic graft preparation. The use of such cells for scientific research is approved by the French authorities in accordance with the regulations in force (authorization DC 2018-3143). Frozen bone marrow samples obtained from patients with AML at diagnosis were selected from an anonymized database registered with the “Commission Nationale de l’Informatique et des Libertés” (the French data protection agency; authorization no. FbP1089790#) and provided by the “Centre de Ressources Biologiques Cancer, Bordeaux Biothèques Santé” at Bordeaux University Hospital. Written informed consent was obtained from each patient for the use of his/her biological samples for research, in accordance with the Declaration of Helsinki. Detailed information about the donors is provided in Supplementary Table S2. This study was performed in accordance with French law (Art. L.1243-1 and Art. L.1245-2 of the French Public Health Code).

## Detailed methods

### Cell preparation and NK cell enrichment

Samples were maintained in liquid nitrogen, transported on dry ice and stored either in liquid nitrogen or at −80 °C if processing was planned shortly after arrival. On the day of processing, samples were thawed and put in RPMI + 20% FCS medium containing DNase I (Roche). Cells were then washed with DPBS + 5% FCS + 2 mM EDTA. Cell viability was assessed with Trypan Blue dye. Cells were then transferred into a 5 mL FACS tube and washed with FACS buffer. NK cells were then enriched by magnetic labeling of the contaminating cells with a Miltenyi Biotec NK cell isolation kit, leading to specific enrichment of unlabeled NK cells.

### NK cell sorting by flow cytometry

After enrichment, cells were washed and incubated at a dilution of 1/50 with mouse serum (Sigma-Aldrich) for 10 min at 4 °C. Cells were stained with anti-CD3 (clone UCHT1, FITC, Beckman Coulter), anti-CD14 (clone RMO52, FITC, Beckman Coulter), anti-CD19 (clone J3-119, FITC, Beckman Coulter) anti-CD45 (clone HI30, APC-Cy7, Biolegend) and anti-CD56 (clone N901(NKH-1), PE, Beckman Coulter) antibodies for 30 min at 4 °C. We added an anti-CD34 (clone 581, FITC, BD Biosciences) antibody to the lineage to sort AML samples. Cells were then washed twice with DPBS and incubated in DPBS with a dead cell marker for 10 min at 4 °C. Cells were washed again and sorted in an Influx or Melody cell sorter from BD (Becton Dickinson, San Diego, USA).

### Single-cell RNA sequencing

After sorting, cells were washed in DPBS + 0.04% BSA, as recommended by the 10x Genomics sample preparation protocol, and kept on ice until counting was performed. We used a 10x Genomics Chromium single-cell 3^’^ v2 kit and protocol to prepare the libraries. HalioDx (Marseille, France) performed RNA sequencing on the NextSeq 500 platform with a sequencing depth of at least 50,000 reads per cell.

### Flow cytometric assessment of the cell surface phenotype

Frozen bone marrow from healthy donors and a set of AML patients different from that used for scRNAseq analysis was thawed, treated with DNAse I (Roche), and stained the following day with antibodies against CD122 (clone Mik-β3, BV786, BD Biosciences), CD127 (clone HIL-7R-M21, Pe-Cy7, BD Biosciences), CD14 (clone M5E2, BUV737, BD Biosciences), CD16 (clone 3G8, BUV737, BD Biosciences), CD160 (clone BY55, Alexa Fluor^®^ 488, BD Biosciences), CD19 (clone SJ25C1, BUV737, BD Biosciences), CD27 (clone M-T271, PerCP-Cy™5.5, BD Biosciences), CD3 (clone UCHT1, BUV496, BD Biosciences), CD45 (clone 2D1, APC-H7, BD Biosciences), CD52 (clone 4C8, Alexa Fluor^®^ 647, BD Biosciences), CD56 (clone B159, Pe-Cy7, BD Biosciences), CD69 (clone FN50, BV605, BD Biosciences), perforin (clone δG9, PE-CF594, BD Biosciences), KIR2DL2/L3/S2 (clone GL183, PE-Cy5, Beckman Coulter), CD45 (clone HI30, APC-Cy7, Biolegend), and KIR2DL1/S1 (clone 11PB6, PE-Vio770, Miltenyi Biotec), and the dead cell marker (BV510, Invitrogen). Samples were analyzed in a Canto II cytometer from BD (Becton Dickinson, San Diego, USA) or on a Fortessa cytometer with UV configuration from BD (Becton Dickinson, San Diego, USA). NK cells were defined as CD3^−^ CD56^+^ or CD3^−^ CD14^−^ CD19^−^ CD56^+^ cells. Statistical significance was assessed by Friedmann’s analysis with Dunn’s post hoc test on paired subset data or with Dunn’s test, and *p* values were adjusted by the Benjamini–Hochberg method. *p value < 0.05, **p value < 0.01, ***p value < 0.001, ****p value < 0.0001.

### Flow cytometry to assess NK cell functionality

Frozen bone marrow from healthy donors and a set of AML patients different from that used for scRNAseq analysis was thawed, treated with recombinant DNAse I (150 U, Roche) and then dispensed the following day into U-bottom 96-well plates at a density of 5 × 10^5^ PBMCs/well. Cells were incubated for 4 h at 37 °C in the presence of Golgi Stop (1/1500; Becton Dickinson), anti-CD107 antibodies (anti-CD107a clone H4A3 and anti-CD107b clone H4B4, FITC, Becton Dickinson), PMA (2 ng/mL) and ionomycin (500 ng/mL). Cells were then washed in PBS supplemented with 2% FCS and 1 mM EDTA and stained by incubation for 30 min at 4 °C in buffer containing an anti-CD3 antibody (clone SK7, PerCP-Cy5.5, Becton Dickinson), an anti-CD56 antibody (Clone N901, APC, Beckman Coulter), and 2% normal mouse serum (Sigma Aldrich). Cells were fixed and permeabilized and were then incubated with 2% paraformaldehyde (PermWash; Becton Dickinson) for 10 min in the dark at room temperature. They were then stained by incubation for 30 min at 4 °C with an anti-IFNγ antibody (clone 4S-B3, PE, Becton Dickinson). Samples were analyzed in a Canto II cytometer from BD (Becton Dickinson, San Diego, USA). NK cells were defined as CD3^−^ CD56^+^ cells. Statistical significance was assessed by Dunn’s test, and *p* values were adjusted by the Benjamini–Hochberg method. *p value < 0.05, **p value < 0.01, ***p value < 0.001, ****p value < 0.0001.

## Quantification and statistical analysis

### Preprocessing of samples

Raw FASTQ files were processed with CellRanger software (v3.0.0 or 3.0.1), which performs alignment, filtering, barcode counting and UMI counting. CellRanger software was used to align reads with the GRch38 genome. Low-quality cells were excluded in an initial quality-control step, which removed genes expressed in fewer than three cells and cells expressing fewer than 200 genes. Cells with less than 10% ribosomal genes in samples from healthy controls and less than 15% ribosomal genes in samples from patients with AML were excluded. We also excluded cells with a UMI count of more than three median absolute deviations from the median value. In total, 23,850 healthy and 15,046 AML cells were retained for further analysis. Library size normalization was performed on the gene expression values for each cell barcode after UMI collapsing, with scaling by the total number of transcripts and multiplication by 10,000. The data were then log-transformed before further downstream analysis with Seurat.^74^

### Sample analysis

We first considered each donor separately. We selected genes with a high variance using the FindVariableGenes function with default parameters. We then reduced the dimensionality of our data by PCA and selected the number of variable PCs retained by random sampling. Cells were clustered with Seurat’s FindClusters function with the Louvain algorithm. We tested a range of cluster resolution parameters and selected the parameter validated by the machine learning approach of Valentine Svensson used in one of our previous studies (http://www.nxn.se/valent/2018/3/5/actionable-scrna-seq-clusters).^12^ We removed cells assigned to clusters accounting for <2% of the cells to avoid doublets, and we also removed a small cluster of cells from two donors that were clustered away from the other clusters and probably resulted from ILC contamination (87 cells from one donor and 32 cells from the other donor). These cells had a high ILC score (as shown below). We also removed a donor-specific subset of 37 cells not found in seven other donors. We checked for the absence of any remaining contamination with the SingleR package.^75^ For visualization, we applied RunTsne and RunUmap to the cell loadings of the previously selected PCs with default parameters to view the cells in two dimensions. We identified cluster markers with FindAllMarkers, with the parameter only.pos set to TRUE, to obtain only upregulated genes as markers of a cluster relative to all other cells. Marker genes were defined as genes with an adjusted *p* value of < 0.05 by the nonparametric Wilcoxon rank-sum test.

### Pooled sample analysis

We pooled samples with the CellRanger aggregate function and subjected them to normalization to equalize the read depth between libraries. We performed regression on the runs to overcome a batch effect that divided human healthy bone marrow NK cell samples into two categories on the basis of processing. We created three different pools of samples: a pool of healthy donor bone marrow NK cells, a pool of bone marrow NK cells from donors with AML and a pool combining these other pools. We then analyzed the pooled samples as described above.

### Assessment of NK cell purity

We checked for ILC contamination in these pools by scoring our cells with the AddModuleScore function (Seurat) and the gene signatures described by^34^ for ILCs and^10^ for NK cells. The ILC signature was obtained from^34^ by selecting the genes from the NK vs ILC1, 2, and 3 comparison that were upregulated in ILCs and for which the *p* value was <0.05. The NK cell gene signature was obtained from^10^ by merging the list of CD56^bright^ CD16^−^ vs CD56^dim^ CD16^+^ upregulated gene with the list of CD56^bright^ CD16^−^ vs CD56^dim^ CD16^+^ downregulated genes, both of which were sorted with an FC of >2. This step resulted in the additional removal of 10 cells from the bone marrow of healthy donors and 41 from the bone marrow of donors with AML.

### Unsupervised hierarchical clustering

We calculated the mean expression levels of genes with differential expression across clusters and performed hierarchical clustering with these values. For all samples, the Euclidean distance was used for samples, genes and clusters.

### Principal component analysis

PC clustering analysis was performed with the Ade4 package on the mean expression level of all genes among clusters. The gene loadings for PC1 and PC2 corresponded to the genes making the largest contributions, which accounted for 20% of the total information for each PC.

### Heatmap

Heatmaps were generated from the scaled expression (log-normalized UMI counts) values for the discriminating gene sets defining each subset, with an adjusted *p* value of <0.05 by the nonparametric Wilcoxon rank-sum test. The color scale is based on the *z*-score distribution.

### Gene annotations

Cell membrane protein, secreted protein and transcription factor annotations were retrieved from public databases (UniProt, MGI, and NCBI for mice and UniProt, GeneCards, and The Human Protein Atlas for humans). Genes encoding transcription factors were defined as such only if “transcription factor activity” was found among the GO annotations for the gene to prevent the confusion of cofactors and coregulators with transcription factors.

### GO enrichment analysis

We performed GO enrichment analysis with BiomaRt^76^ and the GOstats package.^77^ Enrichment scores (*p* values) for selected GO annotations were calculated by a hypergeometric statistical test with a significance threshold of 0.05. The data were plotted as the −log10 *p* values after Benjamini–Hochberg correction. The significance threshold was set at −log10(0.05).

### Comparison with known NK cell subsets

Module scores for CD56^bright^, CD56^dim^, hNK1, hNK2, hNK_Sp1, hNK_Sp2, and hNK_Sp3 gene expression profiles, as defined by^10^ and^12^ were determined with the AddModuleScore function of Seurat for each of our NK cells at the single-cell level. Briefly, the mean expression level of each gene in the defined expression profiles was calculated for each cell, and the aggregated expression for the control gene sets was then subtracted. All analyzed genes were binned on the basis of the mean expression level, and the control genes were randomly selected from each bin. Violin plots were used to assess the distribution of module scores for each NK cell grouped by subset. Statistical significance was assessed by the Wilcoxon rank sum test with continuity correction or by the Kruskal–Wallis test with Dunn’s multiple comparisons test, with *p* value adjustment by the Benjamini–Hochberg method. *p value < 0.05, **p value < 0.01, ***p value < 0.001, ****p value < 0.0001.

### Pseudotime analysis

For bone marrow cells, we performed pseudotime analysis with Monocle 3 (v0.1.3), and the cells were filtered with Seurat, as described above, for each sample individually. We imported Seurat clustering and NK/NKP scoring metadata into the analysis. The starting point of the trajectory was defined as the endpoint of the branch with the highest NKP signature score.

For splenic cells, we used our previously published dataset^12^ and again performed pseudotime analysis with Monocle 3 (v0.1.3). The starting point of the trajectory was defined as the endpoint of the branch with the lowest mature NK cell score, and cluster assignments were retrieved from our previous analysis.

### Definition of the NK cell vs. NKP gene signature

Affymetrix CEL files were analyzed with the limma package (v3.34.9) with RMA normalization. Genes with an adjusted *p* value of <0.01 were considered to be differentially expressed.

### Definition of the canonical vs. adaptive NK cell gene signature

RNA-seq analysis was performed with the DESeq2 package (v1.26.0). Genes with a log2 fold change in expression of >1 and an adjusted *p* value of <0.05 were considered to be differentially expressed.

### Analysis of TCGA data

Analysis of TCGA data was performed with Xena, developed by the University of California Santa Cruz.^78^ The TCGA dataset for acute myeloid leukemia was selected, and the overall survival of patients stratified by the intensity of *CD160* expression was analyzed via the Kaplan–Meier method. The optimal cutoff value for patient stratification is given, and the *p* value indicated in the plot was calculated with the log-rank test.

## Data and software availability

All sequencing data will be deposited in the NCBI GEO repository and will be accessible in GEO. The schematic representation in Fig. S4 was designed with BioRender.

## Supplementary information

Figure S1

Figure S2

Figure S3

Figure S4

Figure S5

Figure S6

Figure S7

Supplementary figure legend

Table S1
