# Supplementary figures and images for: Single-cell profiling reveals the trajectories of natural killer cell differentiation in bone marrow and a stress signature induced by acute myeloid leukemia
Source: Cell Mol Immunol. 2020 Nov 25;18(5):1290–304. doi: 10.1038/s41423-020-00574-8 (PMC8093261; doi:10.1038/s41423-020-00574-8)

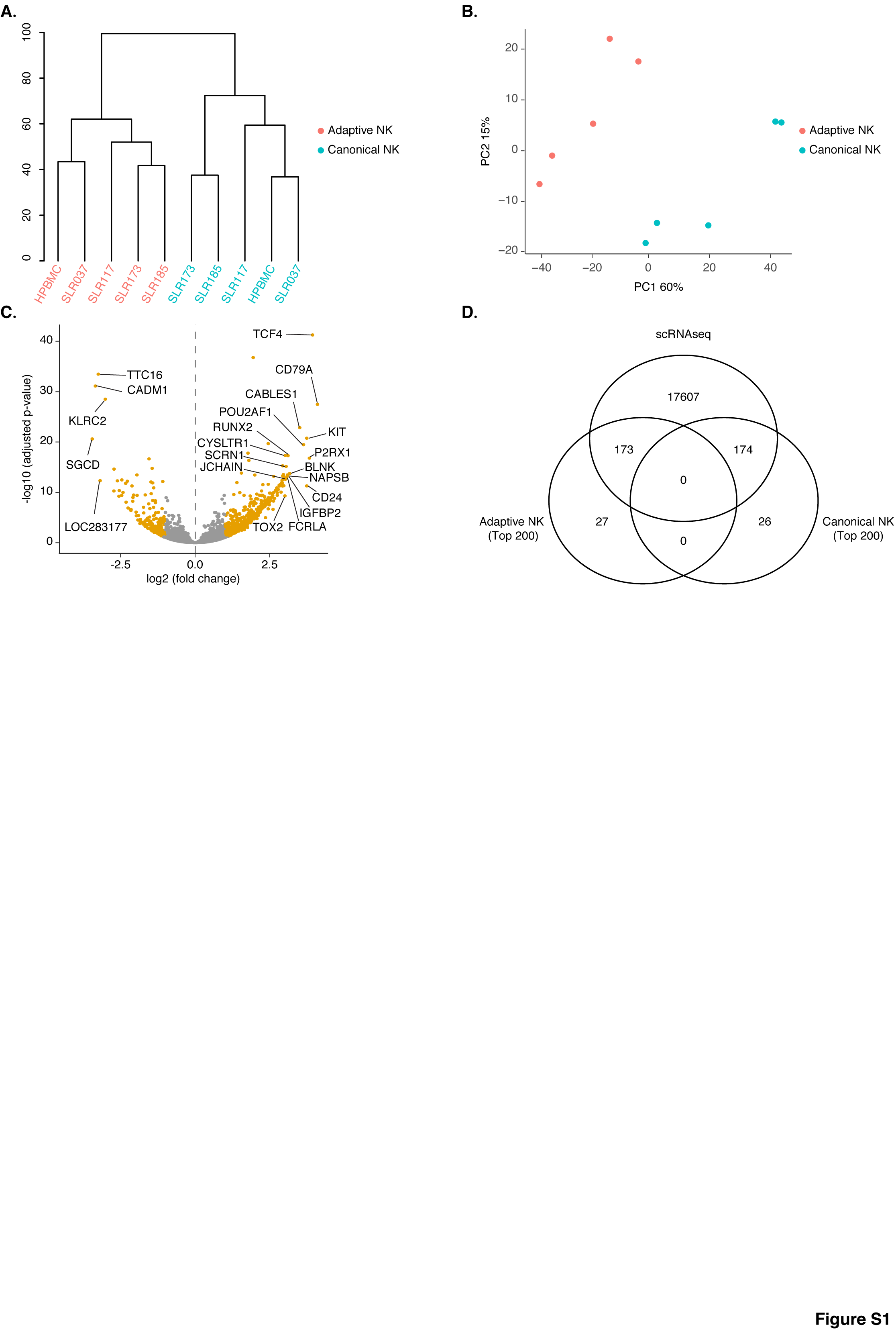

Supplement: Supplementary file 1 — Figure S1 [file 41423_2020_574_MOESM1_ESM.tif]

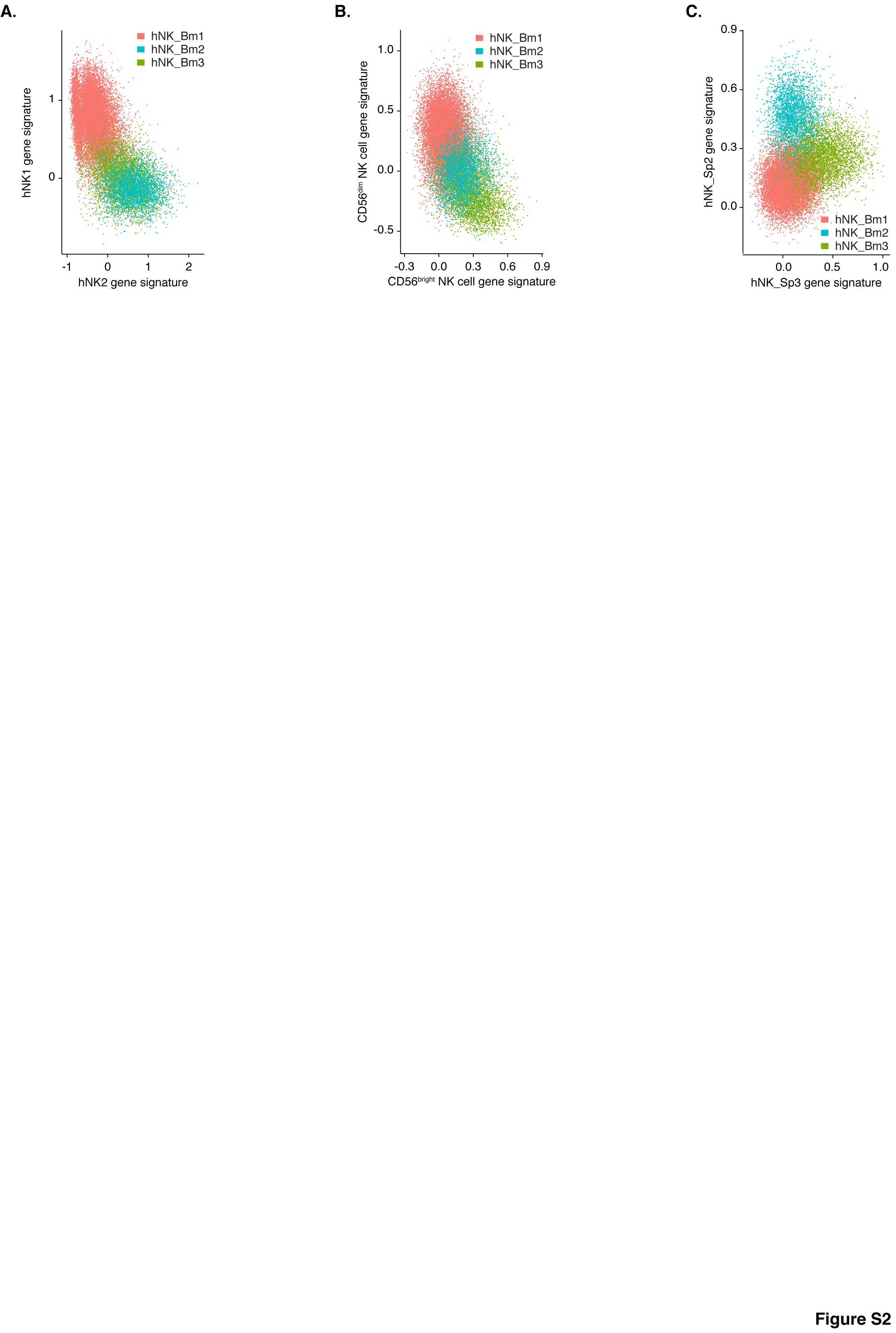

Supplement: Supplementary file 2 — Figure S2 [file 41423_2020_574_MOESM2_ESM.tif]

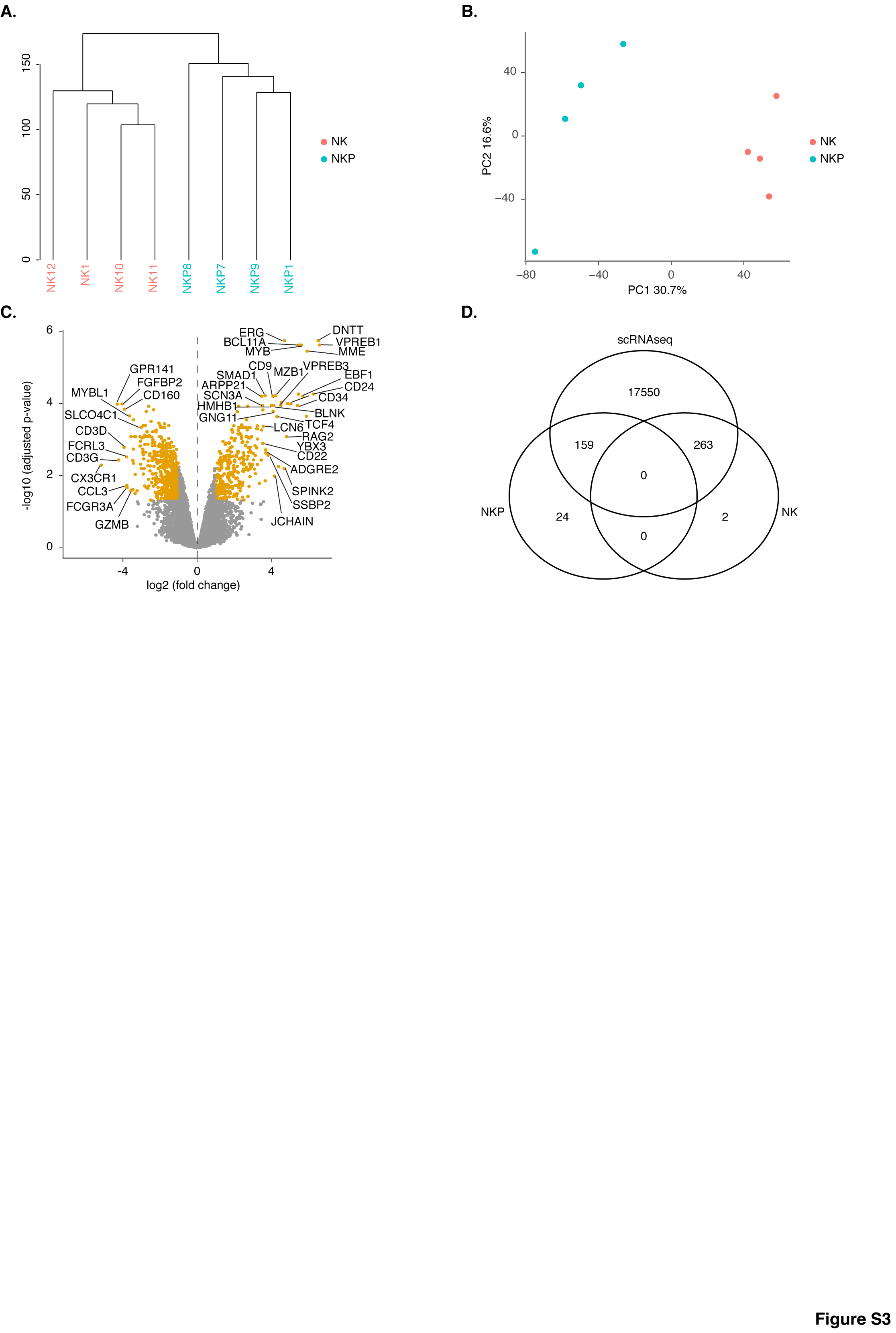

Supplement: Supplementary file 3 — Figure S3 [file 41423_2020_574_MOESM3_ESM.tif]

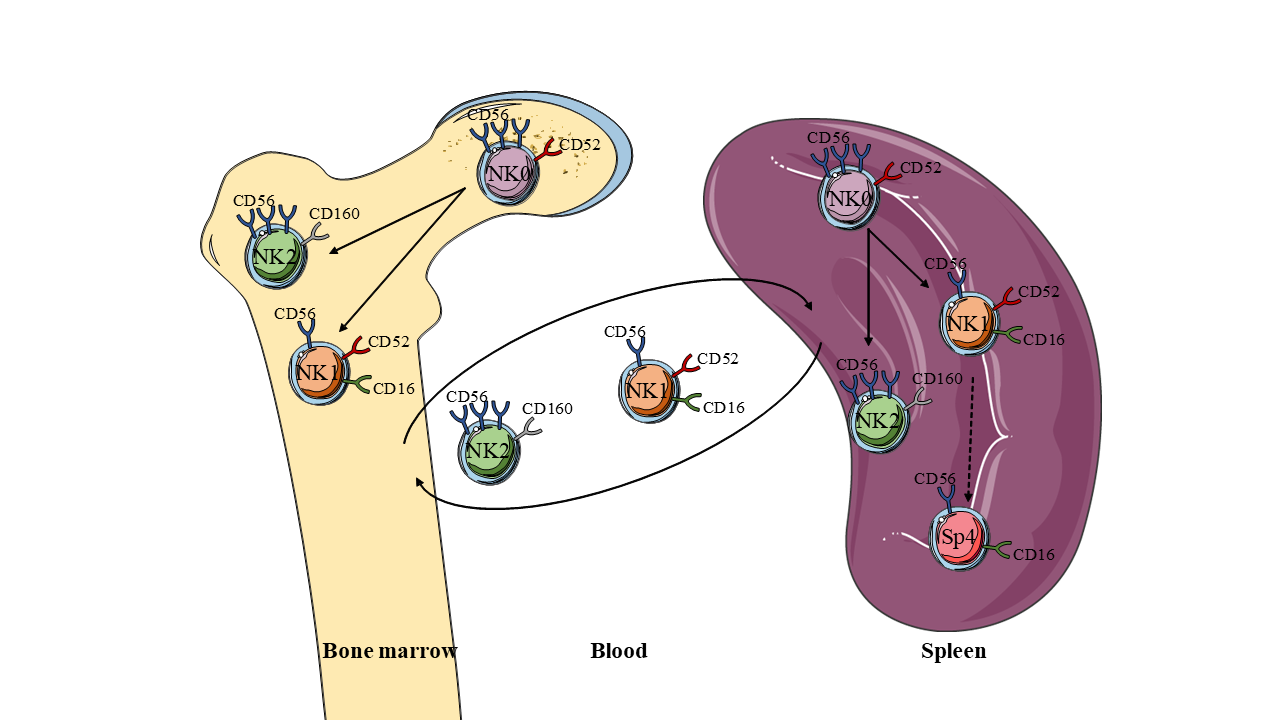

Supplement: Supplementary file 4 — Figure S4 [file 41423_2020_574_MOESM4_ESM.tif]

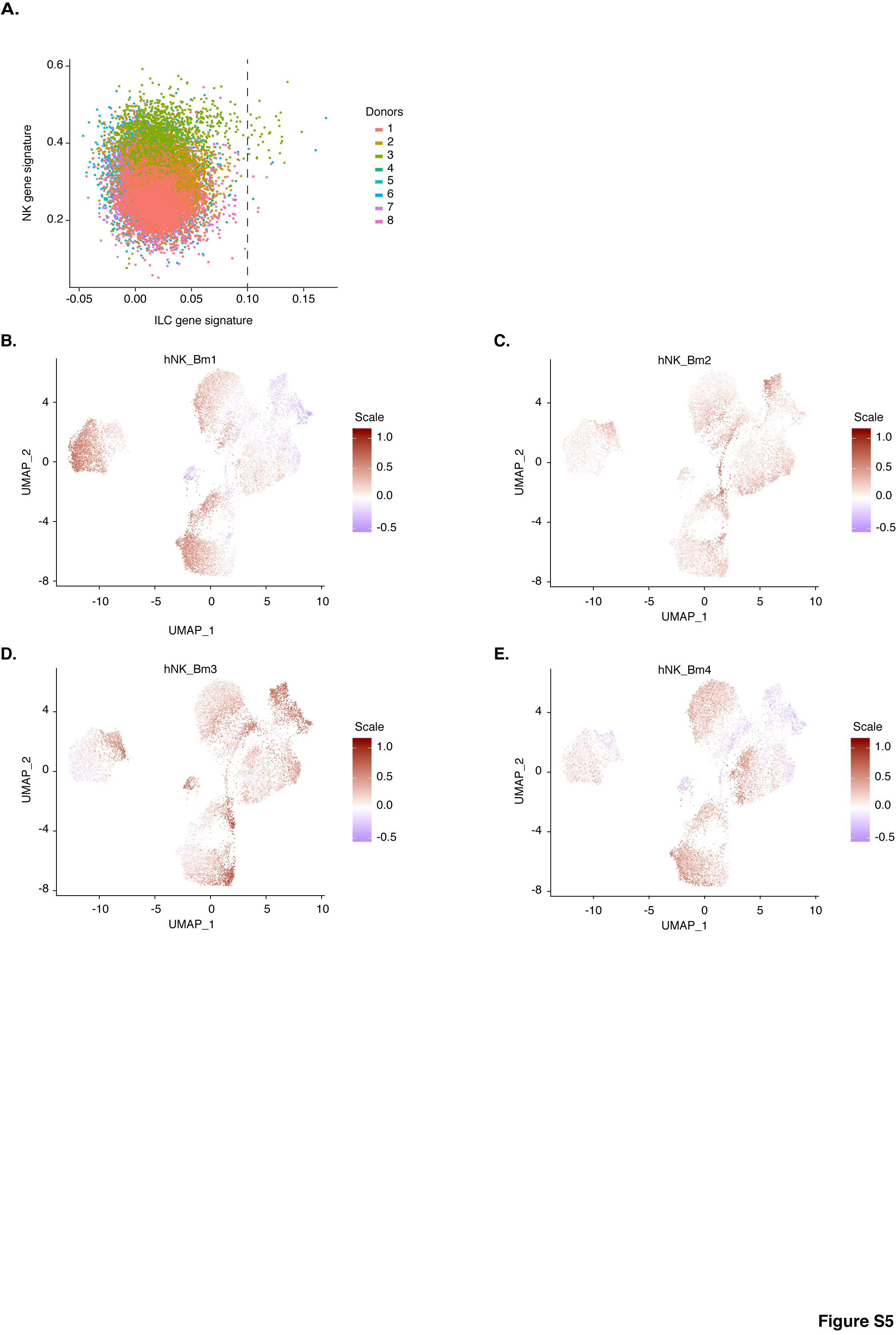

Supplement: Supplementary file 5 — Figure S5 [file 41423_2020_574_MOESM5_ESM.tif]

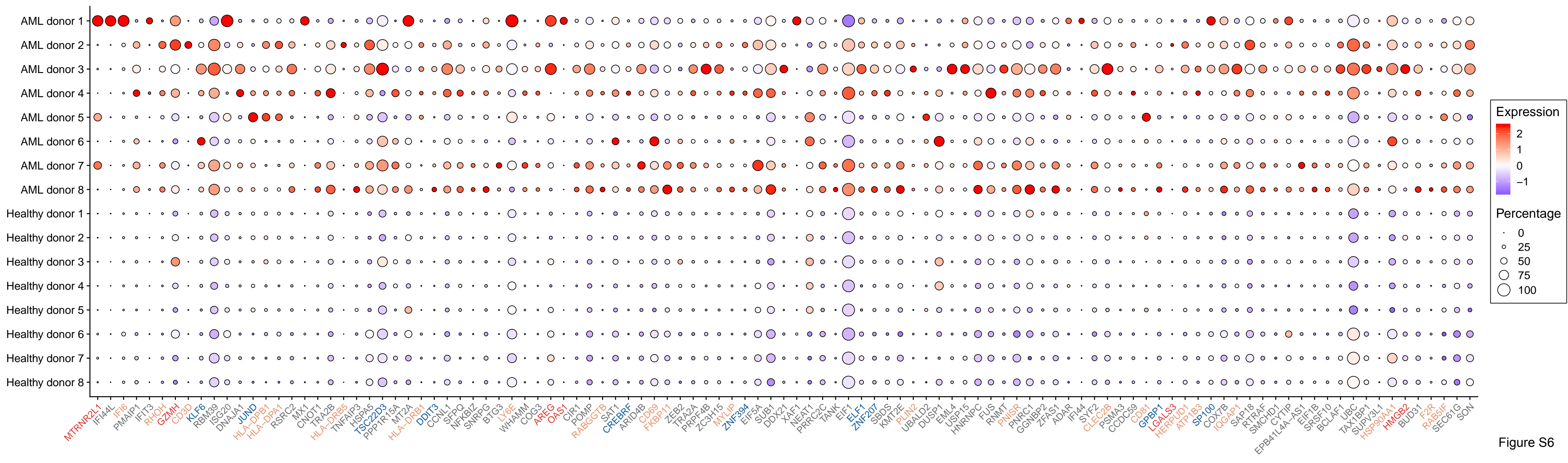

Figure S6

Supplement: Supplementary file 6 — Figure S6 [file 41423_2020_574_MOESM6_ESM.pdf]

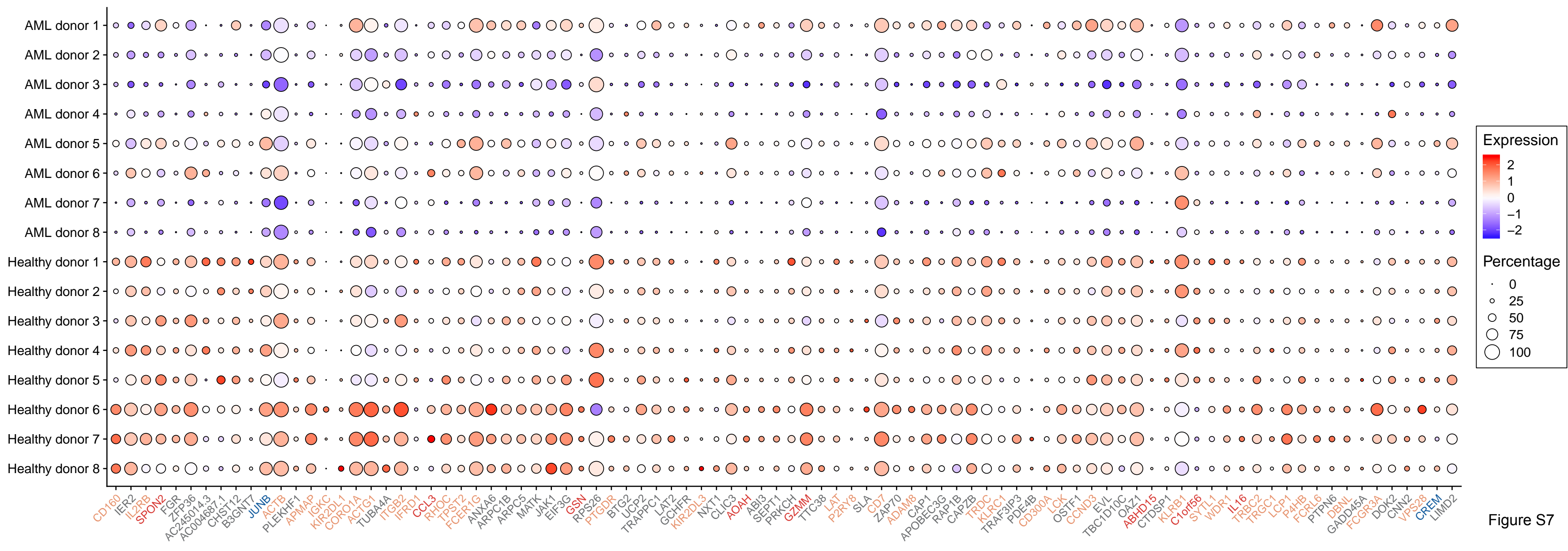

Figure S7

Supplement: Supplementary file 7 — Figure S7 [file 41423_2020_574_MOESM7_ESM.pdf]
