# Supplementary material for: Single-cell profiling reveals the trajectories of natural killer cell differentiation in bone marrow and a stress signature induced by acute myeloid leukemia
Source: Cell Mol Immunol. 2020 Nov 25;18(5):1290–304. doi: 10.1038/s41423-020-00574-8 (PMC8093261; doi:10.1038/s41423-020-00574-8)
Supplement: Supplementary file 8 — Supplementary figure legend [file 41423_2020_574_MOESM8_ESM.docx]

**Figure S1**

**(A)** Unsupervised hierarchical clustering of the 4 replicates of CD56^dim^ CD57^+^ NKG2C^+^ adaptive NK cells and the 4 replicates of CD3^-^ CD56^dim^ CD57^-^ NKG2C^-^ canonical NK cells obtained by re-analysis of the data of Cichocki et al., 2018 [20]. The Euclidean distance between samples is shown. Samples are color-coded according to their relatedness to a particular subset. **(B)** Principal component analysis on 4 replicates of CD56^dim^ CD57^+^ NKG2C^+^ adaptive NK cells and 4 replicates of CD3^-^ CD56^dim^ CD57^-^ NKG2C^-^ canonical NK cells, based on the mean expression level of genes with variable expression. Samples are color-coded according to their relatedness to a particular subset. **(C)** Scatter plot of the genes differentially expressed between CD56^dim^ CD57^+^ NKG2C^+^ adaptive NK cells and CD3^-^ CD56^dim^ CD57^-^ NKG2C^-^ canonical NK cells. Genes displaying significant differential expression (*p*<0.05) are represented by a yellow dot and those with log2(FC)>3 are highlighted. **(D)** Venn diagrams representing the intersection between the genes detected in human bone marrow NK cells from 8 healthy donors by scRNAseq, and the top 200 genes upregulated in CD56^dim^ CD57^+^ NKG2C^+^ adaptive (left) or CD56^dim^ CD57^-^ NKG2C^-^ canonical NK cells (right).

**Figure S2**

**(A)** Module score for the hNK1 and hNK2 NK cell gene expression programs defined by Crinier et al., 2018 [12] for each of the bone marrow NK cells. (**B**) Module score for the CD56^dim^ and CD56^bright^ NK cell gene expression programs defined by Hanna et al., 2004 [10], for each of the bone marrow NK cells. (**C**) Module score for the hNK_Sp2 and hNK_Sp3 NK cell gene expression programs defined by Crinier et al., 2018 [12], for each of the bone marrow NK cells.

**Figure S3**

**(A)** Unsupervised hierarchical clustering of the 4 replicates of CD34^+^ CD38^+^ CD123^-^ CD45RA^+^ CD7^+^ CD10^+^ CD127^-^ NKP and the 4 replicates of CD3^-^ CD56^+^ NKp46^+^ mature NK cells obtained by re-analysis of the data of Renoux et al., 2015 [51]. The Euclidean distance between samples is shown. Samples are color-coded according to their relatedness to a particular subset.

**(B)** Principal component analysis on 4 replicates of CD34^+^ CD38+ CD123^-^ CD45RA^+^ CD7^+^ CD10^+^ CD127^-^ NKP and 4 replicates of CD3^-^ CD56^+^ NKp46^+^ mature NK cells, based on the

mean expression levels of genes with variable expression. Samples are color-coded according

to their relatedness to a particular subset. **(C)** Scatter plot of genes displaying differential expression between CD34^+^ CD38^+^ CD123^-^ CD45RA^+^ CD7^+^ CD10^+^ CD127^-^ NKP and CD3^-^ CD56^+^ NKp46^+^ mature NK cells. Genes displaying significant differential expression (*p*<0.05)

are represented by a yellow dot, and those with log2(FC)>3 are highlighted. **(D)** Venn diagrams representing the intersection between the genes detected in our healthy human bone marrow NK cells from 8 donors by scRNAseq, and the genes upregulated in CD34^+^ CD38^+^ CD123^-^ CD45RA^+^ CD7^+^ CD10^+^ CD127^-^ NKP or in CD3-CD56^+^ NKp46^+^ mature NK cells.

**Figure S4**

Schematic representation of NK0 CD56^bright^ differentiating into NK1 CD56^dim^ and NK2 CD56^bright^ subsets in the bone marrow and spleen. Dichotomous cell surface expression of CD52 and CD160 discriminated the NK0 CD56 ^bright^ from NK2 CD56 ^bright^ subsets. Both NK1 and NK2 were found in the blood. Sp4 appears to be a more mature state of NK1 (Crinier et al.,2018) [12] was found only in the spleen.

**Figure S5**

**(A)** Module score for each of the bone marrow NK cells sorted from 8 different AML donors, for the NK and ILC gene signatures defined by Hanna et al., 2004 [10] and Björklund et al., 2016 [34], respectively. Cells are color-coded according to donor origin. **(B-E)** Module score for the gene expression profiles of the hNK_Bm1 **(B)**, hNK_Bm2 **(C)**, hNK_Bm3 **(D)** and hNK_Bm4 **(E)** subsets in healthy human bone marrow NK cells, for each of our 15,006 AML bone marrow NK cells, on UMAP analysis.

**Figure S6**

Bubble plot representation of genes from the total gene set upregulated in AML bone marrow NK cells relative to healthy bone marrow NK cells. The color scale indicates the intensity of gene expression and circle size is proportional to the percentage of cells expressing the gene in

a given donor. Cell membrane protein genes are color-coded in orange, transcription factor genes in dark blue, secreted protein genes in red and other protein-encoding genes in gray.

**Figure S7**

Bubble plot representation of the genes from the total gene set downregulated in AML bone marrow NK cells relative to healthy bone marrow NK cells. The color scale indicates the intensity of gene expression, and circle size is proportional to the percentage of cells expressing

the gene in a given donor. Cell membrane protein genes are color-coded in orange, transcription

factor genes in dark blue, secreted protein genes in red and other protein-encoding genes in gray.
